# Assessing the potential fire tolerance of conifer saplings in cold and wet environments using a pyro-ecophysiology approach

**DOI:** 10.1186/s42408-025-00443-7

**Published:** 2026-01-13

**Authors:** Alexander S. Blanco, David R. Wilson, Scott W. Rainsford, Grant L. Harley, Roshan P. Bhatta, Corbin W. Halsey, Gabriella M. Eldridge, Daisy P. Estrada Garza, L. May Brown, Madeleine F. Stanley, Jeffrey A. Logan, Aaron M. Sparks, Henry D. Adams, Daniel M. Johnson, Andrew T. Hudak, Li Huang, Alistair M. S. Smith

**Affiliations:** 1 Department of Earth and Spatial Sciences, College of Science, University of Idaho, Moscow, ID 83844 USA; 2 Department of Civil and Environmental Engineering, College of Engineering, University of Idaho, Moscow, ID 83844 USA; 3 Department of Geography, Florida State University, Tallahassee, FL 32306 USA; 4 Northwest Management Inc., , Moscow, ID 83844 USA; 5 School of the Environment, Washington State University, Pullman, WA 99164 USA; 6 Warnell School of Forestry and Natural Resources, University of Georgia, Athens, GA 30602 USA; 7 Rocky Mountain Research Station, United States Forest Service, Moscow, ID 83844 USA

**Keywords:** Mortality, Fire severity, Recovery, Conifer, Fire behavior

## Abstract

**Background:**

Climate change is expected to alter fire return intervals in cold and wet forests in the northwestern United States. This coupled with an expected rise in prescribed fires to restore healthy forests, disproportionately increases risk to saplings of tree species adapted to colder and wetter environments that have low fire resistance. To assess this potential impact, we evaluated the impacts of increasing fire intensity on *Picea engelmannii* and *Thuja plicata* sapling physiology, morphology, and mortality. This was achieved using established pyro-ecophysiology experiments where saplings were subjected to controlled surface fires across a range of fire intensities and post-fire growth, physiology and mortality were assessed up to 7 months post-fire.

**Results:**

In this study we demonstrate that the probability of mortality in the saplings of these two conifer species displays a sigmoidal increase with increasing fire intensity. At fire radiative energy dosage levels < 0.6 MJ m^−2^, the observed mortality in both species was lower than predicted by existing crown scorch-based models due to their limited sensitivity at small diameters. Prior to sapling death, chlorophyll fluorescence transiently recovers before a rapid decline, though the timing varies by species and fire intensity dosage. A new general sapling mortality model derived from 7 conifer species is presented.

**Conclusions:**

Our results provide predictive tools that managers could use to make informed decisions on the potential impacts of fires on conifer saplings growing in cold and wet environments. Results from both species suggest that chlorophyll fluorescence temporal trends could serve as a potential early warning indicator of fire-induced tree mortality, however, future work should explore whether similar responses are observable using remote sensing data from solar-induced chlorophyll fluorescence and assess potential mechanisms underlying this signal. The general sapling mortality model presented in this paper appears to provide an improved method of predicting conifer sapling mortality over existing approaches, however, research is needed to develop coefficients to adjust the model with tree age and environmental factors. Further studies could also explore whether phenotypic plasticity is driving observed tree responses to fire from plants grown from similar environments.

**Supplementary Information:**

The online version contains supplementary material available at 10.1186/s42408-025-00443-7.

## Resumen

**Antecedentes:**

Se espera que el Cambio Climático altere los intervalos de retorno del fuego en bosques fríos y húmedos en el noroeste de los EEUU. Esto, acoplado con una expectativa de incremento de las quemas prescriptas para restaurar la sanidad de los bosques, ha incrementado desproporcionadamente el riesgo de que los renovales de especies forestales se adapten a bosques más fríos y húmedos que tienen menor resistencia al fuego. Para determinar este impacto potencial, evaluamos los impactos de de incrementar la intensidad de los fuegos en la fisiología, morfología, y mortalidad de renovales de *Picea engelmannii* y *Thuja plicata.* Esto fue logrado usando experimentos usuales de piro-ecología en los cuales los renovales fueron objeto de fuegos superficiales controlados a lo largo de un rango de intensidades de fuego y medido su crecimiento en el post fuego, en su fisiología y mortalidad por hasta 7 meses posteriores al fuego.

**Resultados:**

En este estudio demostramos que la probabilidad de mortalidad de los renovales de esas dos especies de coníferas muestra un incremento sigmoide con el incremento de la intensidad del fuego. A niveles de energía radiativa < 0.6 MJ m-2, la mortalidad observada de ambas especies fue menor que la predicha mediante modelos existentes basados en el chamuscado de la corona, debido a su limitada sensibilidad para diámetros pequeños. Antes de la muerte de los renovales, la clorofila fluorescente fotosintética se recupera de manera transitoria antes de una rápida declinación, por lo que ese tiempo varía por especie y por el dosaje en la intensidad del fuego. Se presenta un nuevo modelo general de mortalidad derivado de siete especies de coníferas.

**Conclusiones:**

Nuestros resultados proveen de herramientas predictivas que los gestores pueden usar para informar sobre decisiones de manejo sobre el impacto potencial de los fuegos en los renovales de coníferas creciendo en ambientes fríos y húmedos. Los resultados de ambas especies sugieren que la tendencia de la fluorescencia temporal de la clorofila puede servir como un potencial indicador de alerta temprano de la inducción a la muerte de estos renovales por el fuego; sin embargo, estudios futuros deberían explorar si respuestas similares son observadas usando datos de sensores remotos que usan la fluorescencia de la clorofila derivada de la luz solar y determinar mecanismos potenciales que subrayen esta señal. El modelo general de la muerte de los renovales presentado en este trabajo parece proveer de un método mejorado de predecir la mortalidad de renovales de coníferas sobre las aproximaciones ya existentes. Sin embargo, más investigaciones son necesarias para desarrollar coeficientes para ajustar el modelo con la edad de los árboles y los factores ambientales. Nuevos estudios podrían también explorar si la plasticidad fenotípica puede llevar a obtener las mismas respuestas observadas al fuego en plantas que crecen en otros ambientes similares.

## Background

Climatic and land use change is expected to alter fire return intervals in a wide array of north American ecosystems (Smith et al. 2016a; Bowman et al. 2017; Halofsky et al. 2020; Hessburg et al. 2021). Effects on high elevation forests with cold and wet environments may be complex, resulting in shorter fire return intervals (Rocca et al. 2014; Schoennagel et al. 2017; Halofsky et al. 2020), or possibly longer fire return intervals due to soil dynamics (Krawchuk & Moritz, 2011; Harley et al. 2020). Across the western United States, there is a growing consensus that more fire is needed to maintain healthy ecosystems and reduce wildfire risk (Voelker et al. 2019). Regardless of the cause, shorter fire return intervals will lead to fires disproportionately impacting juvenile trees (i.e., seedlings and saplings). There are several existing models that predict fire-induced mortality in many mature north American tree species (Cansler et al. 2020), yet few studies have focused on juvenile trees (Steady et al. 2019; Sparks et al. 2023; Smith et al. 2025). More knowledge of fire impacts on juvenile trees is needed for land managers to evaluate whether replanting is needed after fires, predict future growth and yield, and understand fire impacts on slope stability (Steady et al. 2019).

**Fig. 1:**
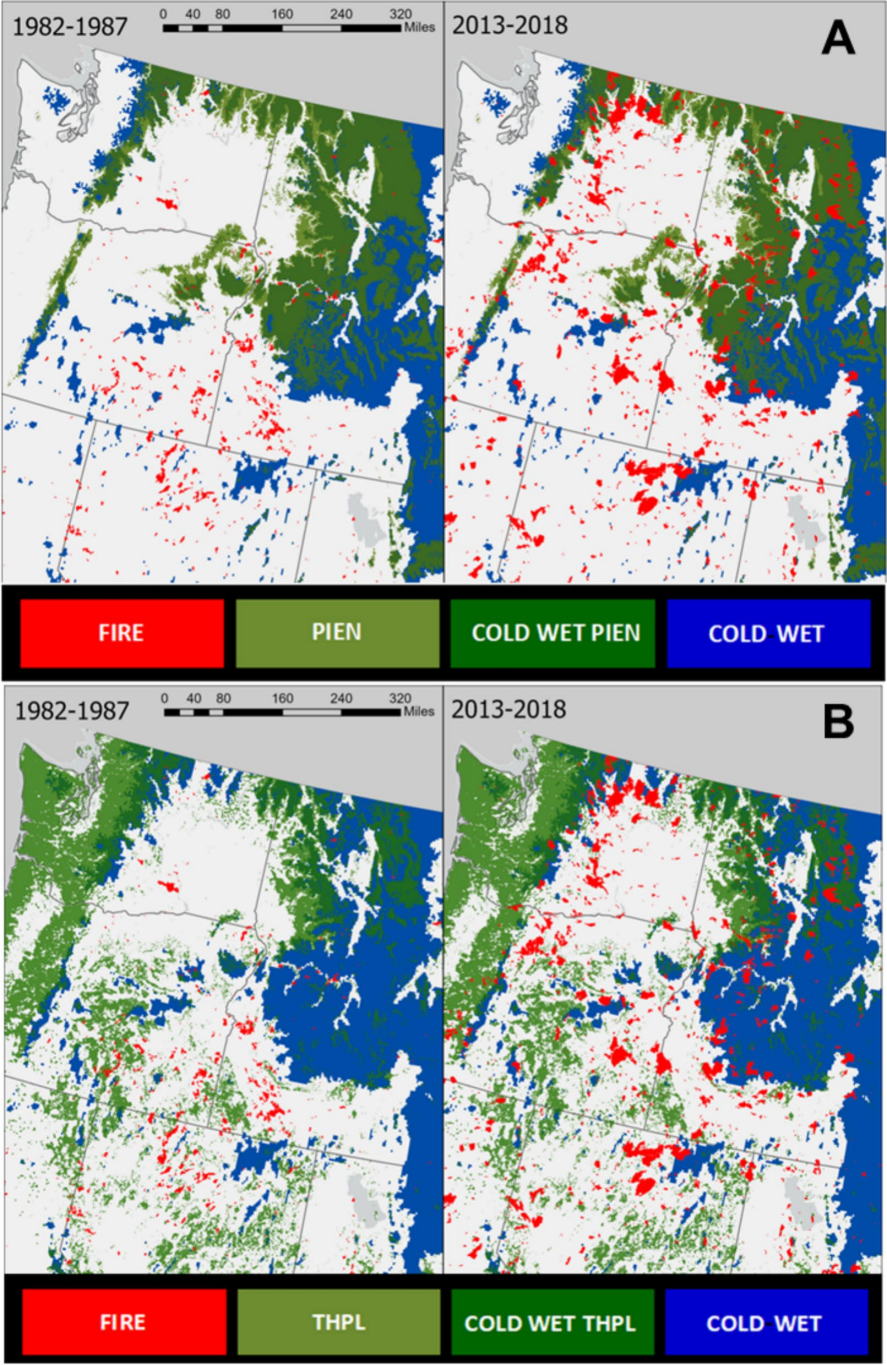
The current range of *P. engelmannii* (**A**) and *T. plicata* (**B**) across the Pacific Northwest, United States with wildfire occurrence and climate variables. Each map displays the current range of PEIN and THPL, the extent of cold and wet climate conditions, and a fire layer indicating areas where fires have been more frequently occurring over time. Sources: Dillon and Gilbertson-Day (2020) and Mathys et al. (2014)

In cold forests at high elevations in the northwestern United States, the seral species Engelmann spruce (*Picea (P.) engelmannii* Parry ex Engel.) grows in areas with sustained snowpacks and in cold valleys from Canada to New Mexico (Alexander 1987; Cooper et al. 1991; Fig. 1A). *P. engelmannii* is used for musical instruments, poles, construction, and pulp (Alexander 1987; Fischer and Bradley 1987; Uchytil 1991). *P. engelmannii* is adapted to long fire return intervals of 150–300 years (Agee 1993) and exhibits low resistance to fires due to thin bark, shallow roots, low branches, and very flammable resin and foliage, often inducing torching fire behavior and immediate mortality. Mature *P. engelmannii* can survive low intensity fires (Starker 1934; Uchytil 1991; Agee 1993), but rigorous experimental research is lacking on the survival of younger *P. engelmannii* in response to low intensity fires (Belote et al. 2015; Hood and Lutes 2017).

Empirical studies consistently report high post-fire mortality for *P. engelmannii*. For example, Starker (1934) reported that only 12% of mature trees were alive five years after fire in some Northern Rocky Mountain stands, a survival rate dramatically lower than that of co-occurring conifers such as western larch (87%) and Douglas-fir (51%). Mortality remains high across a range of fire severities and tree sizes, with basal charring, root injury, and crown damage all contributing to death (Belote et al. 2015). Low-intensity fires confined to the base of the tree can cause mortality in species with limited protective traits (Hood and Lutes 2017; Hood et al. 2018). Because of these challenges, *P. engelmannii* has been a focal species for recent improvements in fire effects models. Species-specific, three-year post-fire mortality models integrated into the First Order Fire Effects Model (FOFEM v5.7 +) incorporated key variables such as crown scorch and cambium kill rating to improve prediction accuracy (Hood and Lutes 2017). While these models represent a notable advance, *P. engelmannii* remains among the most difficult conifers to model accurately due to its extreme fire sensitivity. Despite these advances, there remains a paucity of detailed research specifically targeting fire-related survival outcomes in *P. engelmannii*, particularly under moderate burn severities and in mixed-conifer assemblages. This gap demonstrates the need for additional empirical studies and long-term monitoring to better characterize its fire ecology and improve management strategies in a warming climate (Belote et al. 2015; Hood and Lutes 2017).

In lower-elevation, moist sites, western redcedar (*Thuja (T.) plicata* Donn ex D. Don) occupies a broad ecological range, thriving in maritime climates and nutrient-rich riparian zones throughout the Pacific Northwest and interior wet forests (Fig. 1B). Stands of *T. plicata* typically occur within regions characterized by long fire return intervals, ranging from 50 to 350 years, reflecting their distribution in cool, wet forests with high fuel moisture (Tesky 1992). The species’ wood exhibits exceptional decay resistance and is used in applications ranging from boat building and cabinetry to shingles and utility poles (Minore 1983; Tesky 1992; Klinka and Brisco 2009). Despite its widespread use and ecological importance, *T. plicata* is generally considered fire-sensitive due to thin bark, shallow roots, flammable foliage, and low crown base heights—traits that increase vulnerability to even low- or moderate-severity fires (Minore 1983; Klinka and Brisco 2009; Antos et al. 2016). Post-fire mortality is common, though mature individuals with deeply fluted boles may occasionally survive by effectively elevating critical tissues above the flame zone (Tesky 1992). Still, studies suggest that trees in regions with historically long fire return intervals may exhibit heightened sensitivity, potentially influenced by genetic provenance (Antos et al. 2016).

Recent laboratory research is revealing more nuanced aspects of *T. plicata*'s fire interactions. Fazeli et al. (2022) conducted controlled combustion experiments that exposed live, dried, and dead *T. plicata* needles to convective heating across a range of intensities. *T. plicata* needles exhibited all four phases of combustion (e.g. droplet burning, transition, flaming, and smoldering), each influenced strongly by live fuel moisture content and heat flux. Notably, live needles subjected to high temperatures (≥ 1000 K) ejected and burned ‘intercellular liquid droplets’ within milliseconds of ignition. Fazeli et al. (2022) observed that live western red cedar was ~ 5 times more likely to ignite than dead samples, indicating that live *T. plicata* is highly responsive to variations in heat exposure. This suggests that *T. plicata* is not uniformly flammable across all conditions, and its flammability threshold may be more dynamic than previously thought. In terms of mortality prediction, *T. plicata* also presents challenges for widely used fire effects models. Cansler et al. (2020) evaluated the Ryan and Amman (R–A) model using over 400 tree-level observations from five wildfires. Though the model’s overall performance for *T. plicata* was rated acceptable, it tended to over-predict mortality, particularly in smaller trees and at lower levels of crown volume scorch. As a thin-barked species, *T. plicata* followed patterns seen in other fire-sensitive conifers: high sensitivity and negative predictive value, but low specificity and poor positive predictive value (Cansler et al. 2020). In practice, this means the model was, in general, adequate at predicting which trees would die but frequently misclassified survivors as dead. These limitations are especially pronounced in smaller individuals (DBH < 30 cm), possibly due to underestimation of bark thickness or unmodeled influences like root damage and soil moisture gradients.

Together, recent physiological and modeling research reinforces the view that both *P. engelmannii* and *T. plicata* are highly vulnerable to fire, but while substantial work has been done to characterize mature tree mortality and improve fire effects models for these species, far less is known about how juvenile individuals, particularly saplings, respond to changing fire regimes. This gap is especially relevant as both species inhabit cold, mesic forests where fire return intervals are decreasing, and where sapling cohorts are increasingly exposed to landscape fires. A clearer understanding of the relationships between the heat incident on the trees from fire and the physiological responses in the sapling stage is essential for refining predictive models and informing post-fire management strategies. These needs motivate the following research questions addressed in this study.

1. How does fire intensity affect mortality and physiological response in of *P. engelmannii* and *T. plicata* saplings?
2. What is the dose–response relationship between fire intensity and probability of mortality for each species, and how well do existing mortality models predict observed outcomes?
3. How do these responses compare to those observed in fire-adapted conifers, and what do the differences suggest about fire resistance traits in cold, wet forest species?
4. What are the implications of sapling-level fire impacts for post-fire recovery, replanting, and forest management in colder, mesic ecosystems facing altered fire regimes?

We hypothesized that these two species would be less resistant to fires than species such as *Pinus ponderosa* and *Pseudotsuga menziesii* that are more commonly considered fire resistant, as reflected in higher mortality and physiological stress under increasing fire intensities.

## Methods

### Saplings

For the purposes of this study, we use the term *saplings* rather than *seedlings* to reflect developmental stage, rather than an arbitrary height threshold. While definitions vary, we follow the distinction that seedlings rely primarily on stored seed reserves, whereas saplings are self-sustaining (Good and Good 1972; Thomas and Winner 2002; Brodersen et al. 2019). We purchased 75 T*. plicata* and 75 *P. engelmannii* Parry ex Engel. plants in 3.79-L (1 gallon) pots from *Plants of the Wild* in Tekoa, Washington, USA. Upon arrival at the Washington State University E. H. Steffen Center greenhouse, we transplanted saplings into 6.23-L tree pots (~ 15 × 15 × 41 cm; TP616, Stuewe & Sonc, Inc., Tangent, OR, USA). Pots were filled with a Sunshine mix #4 (Sungro Horticulture, Agawam, MA, USA) and then saplings were grown for 3 months until ready for fire experiments. The saplings were grown following standard procedures (Dumroese 2009) and were well-watered to minimize water stress. At the time of the fire experiment, 42 healthy saplings of each species were randomly selected for use in this study, divided into six treatment groups of n = 7. Prior to the fire intensity experiments, the mean diameter at root collar (DRC) of the *P. engelmannii* saplings was 1.23 ± 0.13 cm, and the mean height was 0.63 ± 0.07 m. The mean diameter at root collar (DRC) of the *T. plicata* saplings was 1.20 ± 0.14 cm, and the mean height was 1.03 ± 0.06 m.

### Replicated fire intensity levels

We follow an established pyro-ecophysiological approach using a toxicological dose–response framework to assess the impacts of increasing fire intensity levels on the morphology and physiology of trees (Kremens et al. 2010; Smith et al. 2016b, 2017), where this approach uses the standard nomenclature associated with dose–response experiments (Table 1). Pyro-ecophysiology has been described as (Smith et al. 2017, 2025; Jolly and Johnson 2018), “*the study of how fire, within its environment, mechanistically interacts with the physiology of an organism*”. Pyro-ecophysiology research has focused on improving a mechanistic understanding of flammability properties across scales (Jolly and Johnson 2018; Jolly et al. 2025), physiological mechanisms associated with damage, mortality, and recovery from heat transferred from fires to plants (Smith et al. 2017; Sparks et al. 2023), and how these mechanisms vary across scales and functional types (Schwilk et al. 2025; Smith et al. 2025).

**Table 1:** Dose–response nomenclature adapted for fire impacts on plants. Adapted from Calabrese and Baldwin (2003a,b)

Acronym	Full term	Description in terms of fire effects on plants
NOAEL	No observed adverse effect level	Fire intensity level where no adverse morphological or physiological impact is observed (e.g., death, growth changes). This does not include visible signs of scorch
LOAEL	Lowest observed adverse effect level	Fire intensity level where an adverse morphological or physiological impact is observed (e.g., death, growth changes)
LD50	Level where 50% mortality occurs	Fire intensity level that on average leads to 50% of death in the plants. Can be defined via immediate or delayed mortality
LD100	Level where 100% mortality occurs	Fire intensity level that on average leads to death in all the plants. Can be defined via immediate or delayed mortality
LDTC50	New term	Fire intensity level that on average leads to 50% of top kill in plants
LDTC100	New term	Fire intensity level that on average leads to 100% top kill in all the plants

**Fig. 2:**
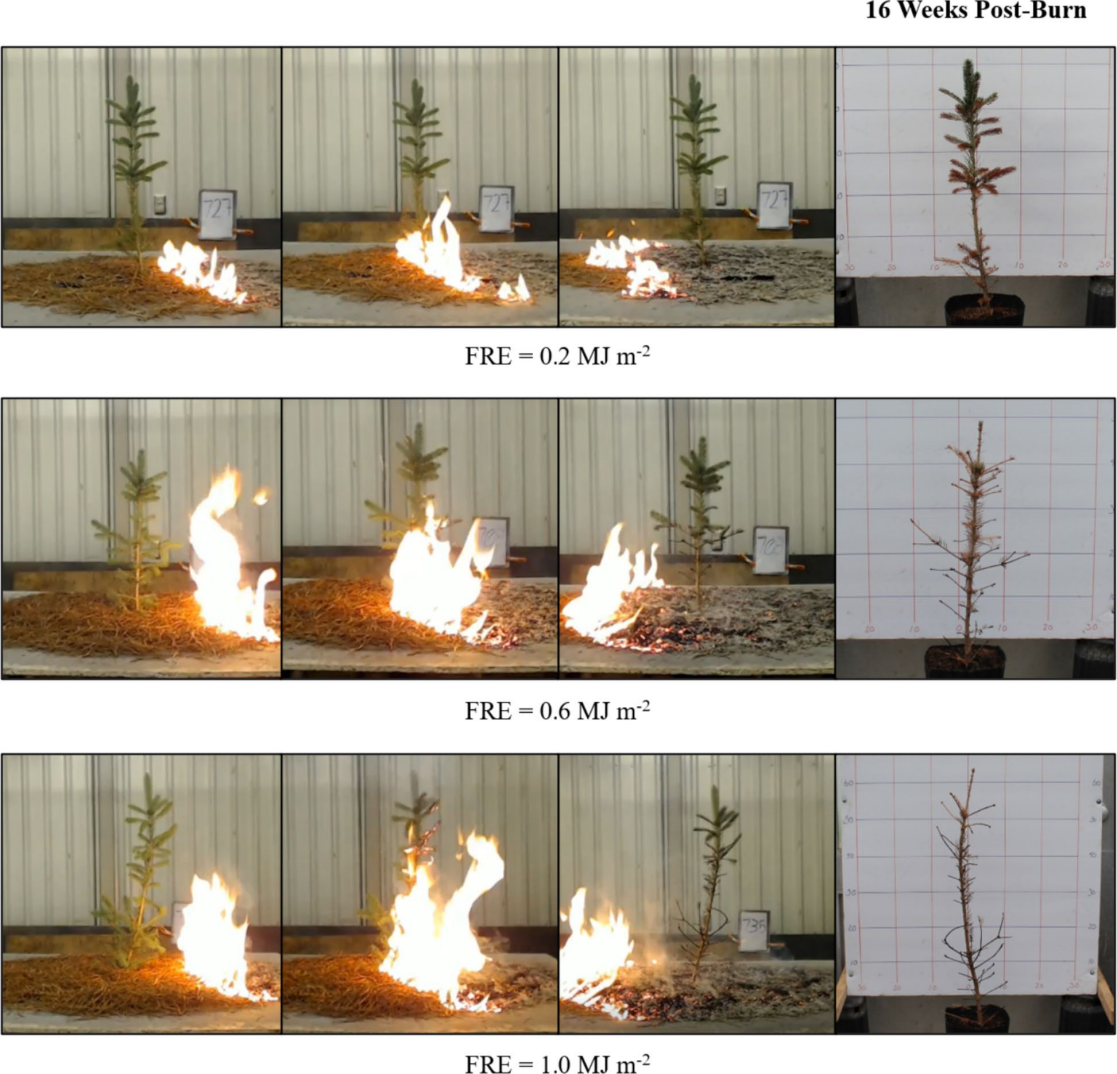
Video images of one representative *P. engelmannii* sapling for the Fire Radiative Energy (FRE) dosage levels of 0.2, 0.6, and 1.0 MJ m^−2^ showing the progression of each experimental burn. Other levels not shown to aid in visual comparison. The last image in each row displays the condition of each sapling at 16 weeks following the fire. Note that the greater FRE dosage level visually corresponds to taller flame height and increased crown mortality after burning

The fire experiments were conducted at the Idaho Fire Initiative for Research and Education (IFIRE) laboratory located in Moscow, Idaho (Fig. 2). This facility consists of an indoor combustion environment, with a separate space for sample preparation and measurements. The facility is located approximately 8 miles from the Steffen Center greenhouses and saplings were transported to and from the combustion facility in an enclosed vehicle. Following prior studies (e.g., Smith et al. 2017; Steady et al. 2019; Sparks et al. 2023, 2024), we subjected saplings to surface fires across a range of fire intensity levels associated with discrete fire radiative energy dosage levels (FRE, MJ m^−2^, Wooster et al. 2021).

The fuel for this study was western white pine (*Pinus monticola*) needles and were collected by raking the forest floor of the R.T. Bingham White Pine Seed Orchard located on the outskirts of Moscow Idaho. The raked pine needles are then manually sorted to remove all bark, twigs, and debris. Following sorting, the pine needles were oven-dried for at least 48 h at 105 °C to achieve near 0% moisture content (Matthews 2010). We then used known fuel loads of the ~ 0% moisture content western white pine needles and the relationship presented by Smith et al. (2013): fuel load = FRE/2.679 to create FRE stratification levels of 0 (unburned), 0.2, 0.4, 0.6, 0.8, and 1.0 MJ m^−2^. The *Pinus monticola* needles were used as they produce consistent and repeatable rates of energy released as a function of biomass consumed, enabling stratified FRE dosage levels to be achieved (Smith et al. 2013). FRE is used as our stratification parameter as although maximum fire radiative power has been shown to be a good predictor of physiological impacts of fire on mature trees (Sparks et al. 2017), it cannot be readily predicted in magnitude or timing making it difficult to be used to plan experimental treatments. Each sapling was then burned separately to avoid pseudoreplication (Steady et al. 2019). Following combustion, we returned saplings to the greenhouses and monitored them for 28 weeks, which included green-up after the following winter. The saplings were watered to field capacity prior to burning to limit impacts of water stress.

### Measurements of plant morphology and physiology

**Fig. 3:**
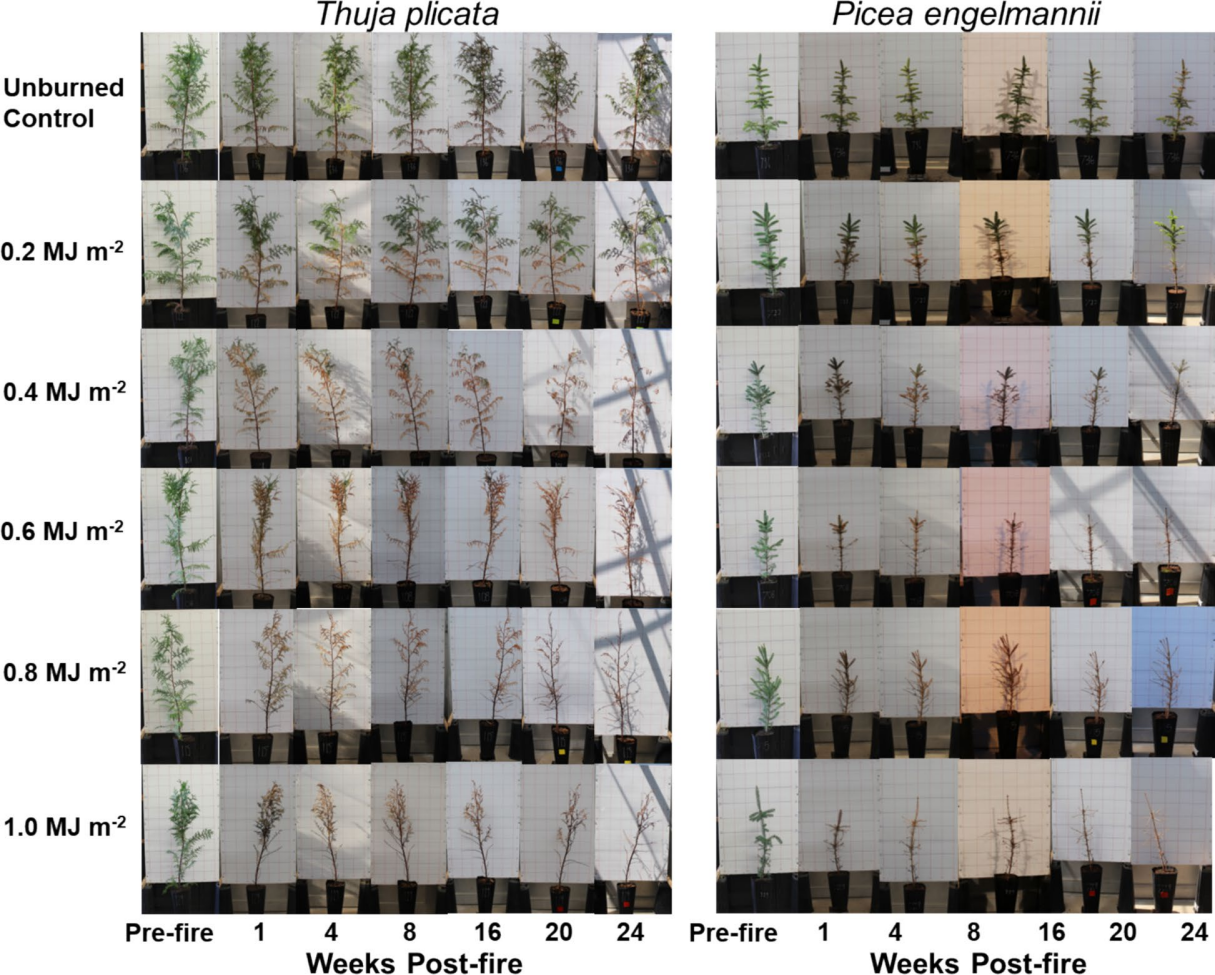
Temporal evolution of sapling morphology of *T. plicata* and *P. engelmannii* saplings for 24 weeks following the fire experiments as shown by the FRE stratification level. Each series of images tracks the temporal evolution of a single representative sample randomly selected from the 7 replicates per treatment. Photographs were collected for all replicates per treatment at all dates (not shown)

For each sapling, we made morphology and physiology measurements within two hours before and after each experimental fire. Durning the experiment, individual burns were record on a Logitech HD Pro Wecam C920 video camera (Logitech International, Newark, USA) located inside the combustion area of the IFIRE Labatory. Post-fire measurements were then taken weekly for the first 4 weeks, at 8 weeks, and every 4 weeks from week 16 to week 28 (Fig. 3). Following Steady et al. (2019), we assessed mortality at 28 weeks using the stem cambium scratch test, where living cambium is green and dead cambium is brown. At each measurement interval, data collected included photographs, sapling height measured with a tape, visual estimation of percentage green crown by volume (PCG), averaged base diameter (accounting for the slight ellipsoidal shape of the bole) measured with a digital caliper (REXBEXI, Auburn, USA), and chlorophyll fluorescence using an OS30P + handheld chlorophyll fluorometer (Opti-Sciences, Hudson, NH, USA). The percentage of canopy volume scorched, c, was estimated as: c = 100%—PCG at 1 week following the fire. All collected data is available in supplemental tables (Tables S1 to S10).

Following Hood and Lutes (2017), we then calculated the probability of fire-induced tree mortality (P_M(PIEN)_) of *P. engelmannii* as:

1 $${P}_{M(PIEN)}=\frac{1}{1+{exp}^{(-\left(0.0845+0.0445*c\right))}}$$

Following Rebain (2022), we then calculated the probability of fire-induced tree mortality (P_M(THPL)_) of *T. plicata* as:

2 $${P}_{M(THPL)}=\frac{1}{1+{exp}^{(-\left(-1.941+6.316(1-{\mathrm{exp}}^{-\mathit{BT}})-0.000535*{c}^{2}\right))}}$$

2b $${BT}_{THPL(R)}=0.025*DOB(inches)$$

where, BT is bark thickness and c is the crown volume scorched as defined above.

Following Ryan and Reinhardt (1988), we also calculated the probability of fire-induced tree mortality of each species as:

3 $${P}_{M(species)}=\frac{1}{(1+{exp}^{\left({b}_{0}+{sp}_{i}+{b}_{1}BT+{b}_{2}{BT}^{2}+{b}_{3}{c}^{2}\right)})}$$

where, for *T. plicata*, sp_i_ = 0.8860 and for *P. engelmannii,* sp_i_ = −1.495 (Ryan and Reinhardt 1988). In each case, the coefficients are (Ryan and Reinhardt 1988): b_0_ = −0.9245, b_1_ = 0.9407, b_2_ = −0.0690. and b_3_ = −0.00542. For Eq. (3), BT is calculated following the relations of Smith and Kozak (1967) as reported in Ryan and Reinhardt (1988):

3b $${BT}_{THPL(SK)}=0.386+(0.021*DOB)$$

3c $${BT}_{PIEN(sk)}=0.189+(0.022*DOB)$$

where DOB denotes diameter outside bark (cm), which in this study is approximated by the DRC.

Following Sparks et al. (2023), we made chlorophyll fluorescence measurements at least 1 h after sunset so that the needles could dark-adapt. For each of the saplings, the minimum fluorescence (F_o_) was measured, and the maximum fluorescence (F_m_) was then measured after a short saturation pulse of (3500 μmolm^−2^ s^−1^) of red light centered at 660 nm. Following Gentry et al. (1989), the maximum quantum yield of photosynthesis II or F_v_/F_m_ was calculated by:

4 $$\frac{{F}_{V}}{{F}_{M}}=\frac{{F}_{m}-{F}_{0}}{{F}_{M}}$$

Following Sparks et al. (2018, 2023), Differences between treatment groups were compared with ANOVA and, if significant (α = 0.05), a Tukey’s honest significant difference test.

## Results

**Fig. 4:**
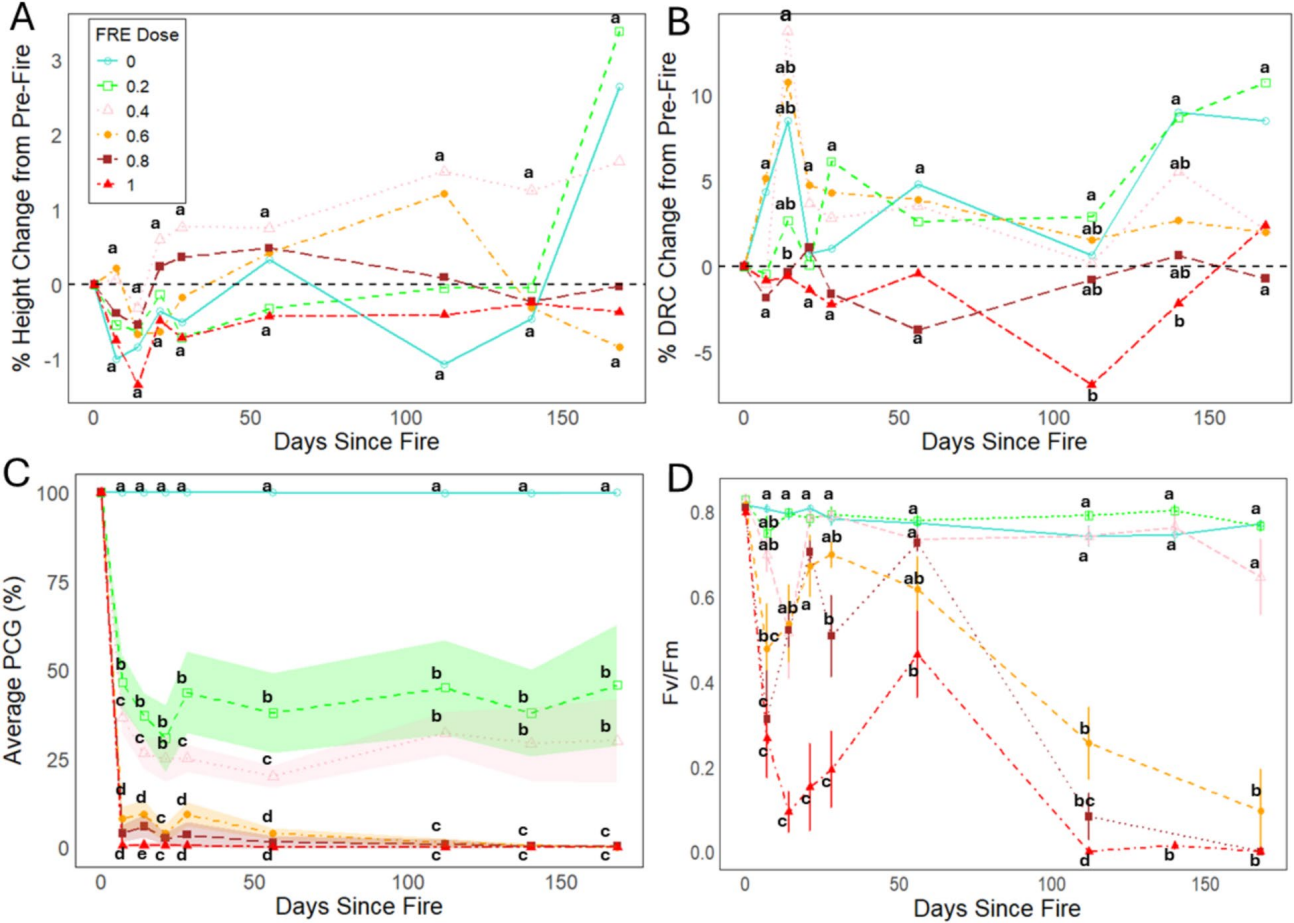
Plant physiology, morphology, and mortality responses due to increasing Fire Radiative Energy (FRE) for *P. engelmannii* saplings. Superscript letters denote statistical differences among treatments within species. Data are shown for (**A**) percentage change in sapling height, (**B**) percentage change in diameter at root collar (DRC), (**C**) percentage change in crown that is green (PCG) with shaded regions representing a 95% confidence interval, (**D**) dark-adapted leaf photochemical efficiency expressed as leaf chlorophyll fluorescence (Fv/Fm),

**Fig. 5:**
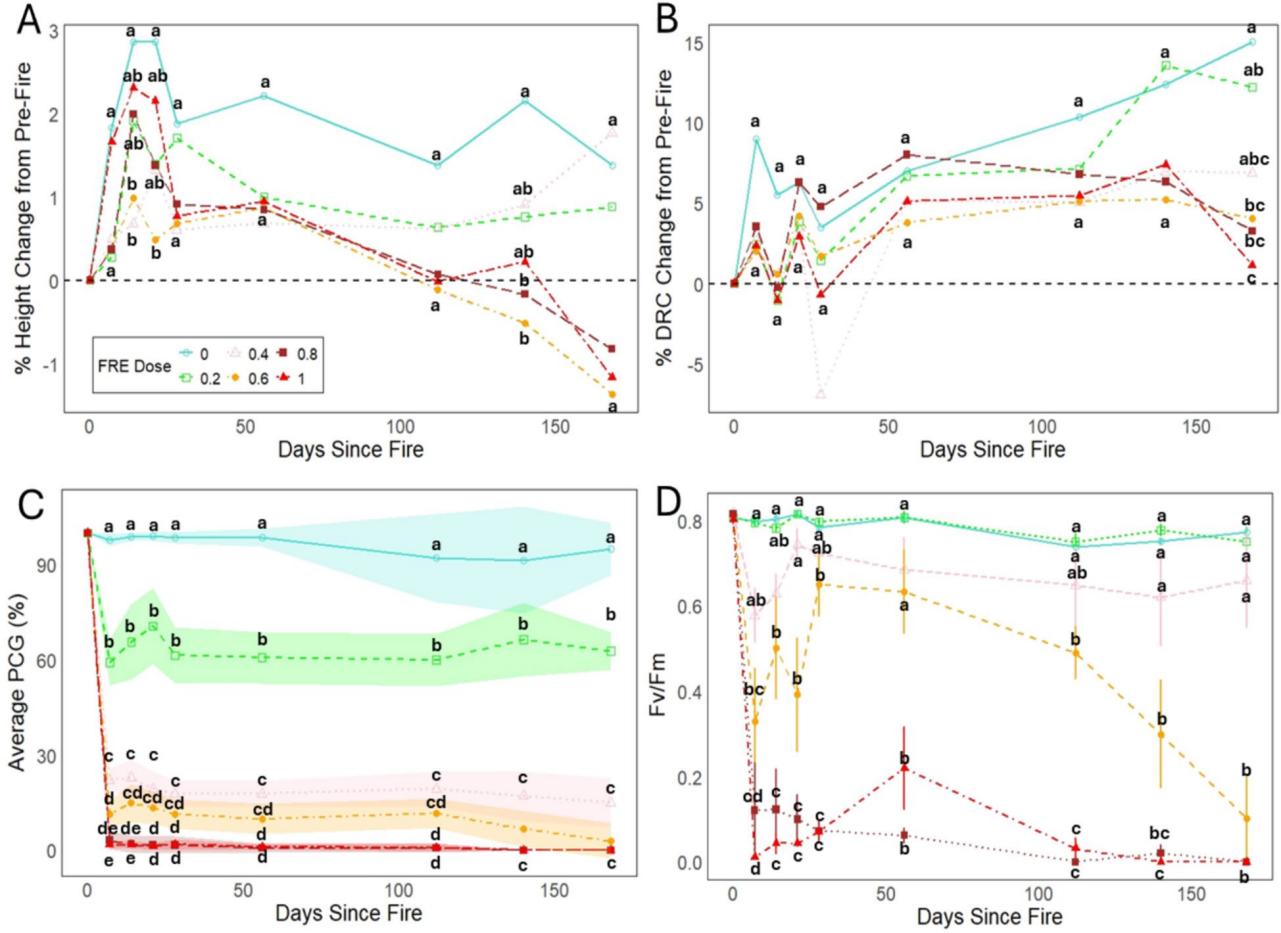
Plant physiology, morphology, and mortality responses due to increasing Fire Radiative Energy (FRE) for *T. plicata* saplings. Superscript letters denote statistical differences among treatments within species. Data are shown for (**A**) percentage change in sapling height, (**B**) percentage change in diameter at root collar (DRC), (**C**) percentage change in crown that is green (PCG) with shaded regions representing a 95% confidence interval, (**D**) and dark-adapted leaf photochemical efficiency expressed as leaf chlorophyll fluorescence (Fv/Fm)

Post-fire height change was generally lower in saplings subjected to higher FRE doses, however there was no significant difference in height change across the treatment groups of both species (Fig. 4A, Fig. 5A). Similarly, for *P. engelmannii* we observed no significant difference across treatment groups for change in diameter at most time steps post-fire (Fig. 4B). In contrast, at 28-weeks post-fire we observed a clear dose–response associated with the *T. plicata* sapling DRC change, with the treatment levels divided into three distinct groups, where the break points occurred at 0.6 MJ m^−2^ and 1.0 MJ m^−2^ (Fig. 5B). In both the *P. engelmannii* and *T. plicata* saplings we observed a clear dose–response in PCG, with three statistically significant (*p* < 0.05) groups observed for each species. For *P. engelmannii* the breakpoints occurred at 0.2 MJ m^−2^ and 0.6 MJ m^−2^ (Fig. 4C) while for *T. plicata* the breakpoints occurred at 0.2 MJ m^−2^ and 0.4 MJ m^−2^ (Fig. 5C). The photographic temporal series (Fig. 3) illustrates that in each species notable crown damage was apparent 1-week post-fire across all fire intensity dose levels above the unburned control, but that the severity of crown damage clearly increased with both time and fire intensity level. Each photographic temporal series was associated with one of the seven samples randomly selected for this treatment.

### Chlorophyll fluorescence dose–response

In both the *P. engelmannii* and *T. plicata* saplings we observed two significantly different chlorophyll fluorescence groups (*p* < 0.05), with a consistent threshold identified at 0.6 MJ m⁻^2^ for both species (Fig. 4D and Fig. 5D). For both species, fire intensities above 0.4 MJ m⁻^2^ triggered a rapid decline in chlorophyll fluorescence followed by a partial recovery. At or above 0.6 MJ m⁻^2^, this recovery was only temporary and preceded a second sharp decline. In the *P. engelmannii* saplings this false recovery peak occurred 4-weeks post-fire for the 0.6 MJ m^−2^ level and at 8-weeks post-fire for the 0.8 MJ m^−2^ and 1.0 MJ m^−2^ levels (Fig. 4D). In the *T. plicata* saplings, we observed a similar temporal trajectory with the 0.4 MJ m^−2^ to 0.6 MJ m^−2^ levels peaking at 4-weeks post-fire and the 1.0 MJ m^−2^ level peaking at 8-week post-fire. In contrast to *P. engelmannii*, the *T. plicata* saplings did not exhibit a clear recovery in chlorophyll fluorescence at the 0.8 MJ m^−2^ level.

### Mortality dose–response

**Fig. 6:**
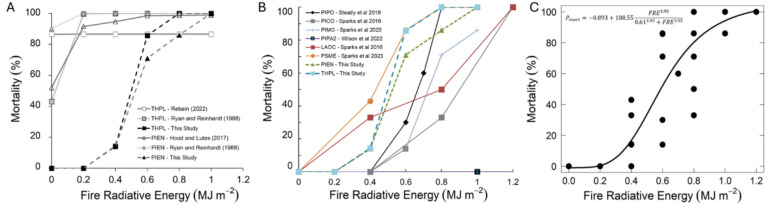
**A** Dose–response curve of sapling mortality versus FRE (fire radiative energy) dosage using data from the current study and the relationship presented by Ryan and Reinhardt (1988), Hood and Lutes (2017), and Rebain (2022). **B** = Cross-comparison of FRE versus mortality for conifer species assessed to date. **C** General predictive relationship derived from FRE to mortality dose–response data for 7 conifer species. Species are indicated with their United States Department of Agriculture Natural Resources Conservation Service symbols, PIPO: *Pinus ponderosa*, PICO: *Pinus contorta.* var. *latifolia*, PIMO: *Pinus monticola*, PIPA2: *Pinus palustris*, LAOC: *Larix occidentalis*, PSME: *Pseudotsuga menziesiil*, THPL: *Thuja plicata*, and PIEN: *Picea engelmannii*

Fire-induced mortality increased with fire intensity following a sigmoidal form (Fig. 6), with all saplings surviving at the 0.2 MJ m⁻^2^ treatment level and the 100% lethal dose (i.e., LD100) occurring at 0.8 MJ m^−2^ and 1.0 MJ m^−2^ for the *P. engelmannii* and *T. plicata* saplings, respectively. Comparison of observed mortality with predictions from the crown-scorch models (Eqs. 1, 2 and 3) demonstrates that all three models overestimate mortality at low to moderate fire intensities (< 0.6 MJ m⁻^2^).

**Fig. 7:**
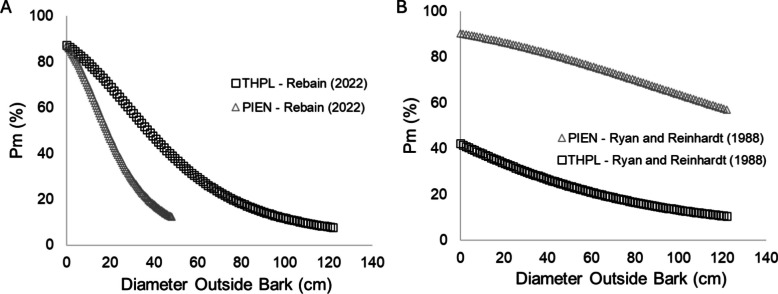
Sensitivity analysis of (A) Eq. 2 and (**B**) Eq. 3 as a function of diameter outside bark. Sensitivity analysis as a function of bark thickness and crown volume scorched were previously presented in Rebain (2022) and Ryan and Reinhardt (1988). In each case, crown volume scorched was kept constant at zero and Pm(%) denotes the percentage mortality. The y-intercepts confirm the approximate modeled mortality for the control (unburned) small diameter stems in Fig. 6. Species are indicated with their United States Department of Agriculture Natural Resources Conservation Service symbols, THPL: *T. plicata* and PIEN: *Picea engelmannii*

Given Eq. (1) is only dependent on c, values of zero (unburned) yield a constant of Pm = 0.52 and therefore this model is insensitive to changes in bark thickness or tree diameter outside bark. In terms of Eqs. (2) and (3), sensitivity analysis as a function of bark thickness and crown volume scorched were presented in Rebain (2022) and Ryan and Reinhardt (1988), respectively. Consequently, in Fig. 7A and Fig. 7B, we illustrate this same analysis in terms of diameter outside bark for c = 0. Sensitivity analysis of Eq. (2) demonstrates that values of c = 0 yield a LD50 at a diameter outside bark of < 38 cm and < 18 cm for *T. plicata* and *P. engelmannii*, respectively. Sensitivity analysis of Eq. (3) demonstrates that *T. plicata* exhibits a lower Pm (~ 42%) at c = 0, while *P. engelmannii* yields a LD50 at a diameter outside bark of < 148 cm. Taken together, the projected high mortality at low diameter outside bark explains the overestimation of mortality observed in Fig. 6B.

## Discussion

In contrast to the results of Steady et al. (2019), increases in fire intensity level did not produce a step-wise response to percentage changes in sapling height (Fig. 4A and Fig. 5A) or DRC (Fig. 4B and Fig. 5B), which may be due to saplings being burned too late in the growing season. The lack of a FRE response associated with fire radiative energy may also be due to other heat transfer modes such as convection or conduction being more dominant, like what was observed on older *Pinus ponderosa* trees by Sparks et al. (2017) in landscape-scale fires. Consistent with previous experiments assessing the impacts of increasing fire intensity on other conifer species (e.g., Bowman et al. 2018; Steady et al. 2019; Sparks et al. 2023) we observed a clear sigmoidal (s-curve) relationship between FRE and the probability of sapling mortality*.* In Fig. 6B, we compare our data with other studies that also used FRE (Sparks et al. 2016, 2023; Smith et al. 2017; Steady et al. 2019; Wilson et al. 2022). Although not equivalent to FRE, a study assessing fire temperatures at 5 cm height also showed similar sapling mortality s-curves when considering conifer sapling responses to fire in *Callitris intertropical* (Bowman et al. 2018).

Notably, both *P. engelmannii* and *T. plicata* exhibited 100% survival at 0.2 MJ m⁻^2^ and demonstrated fire resistance equal to or exceeding that of *Pseudotsuga menziesii* at FRE levels ≥ 0.6 MJ m⁻^2^. This result is surprising given that *P. engelmannii* and *T. plicata* are widely considered some of the most fire-vulnerable conifers in the northwestern United States, while *Pseudotsuga menziesii* is considered one of the most fire-resistant tree species (after *Pinus ponderosa*) in the region (Starker 1934; Fischer and Bradley 1987; Tesky 1992; Agee 1993). Although Fischer and Bradley (1987) mainly focused on mature trees, they remarked that *P. engelmannii* is highly susceptible to fire-induced tree mortality because of flammable foliage and thin bark, while *T. plicata* also has been characterized as having a high to moderate susceptibility to fire-induced tree mortality because of thin bark and low-lying branches.

Our results suggest that interspecific differences in fire tolerance do not manifest in saplings as they do in mature trees, as saplings have not yet developed the thick bark and other traits typical of fire-resistant mature trees (Agee 1993; Smith et al. 2018; Pausas 2018). It is also worth noting that the current understanding of interspecific fire vulnerability is based entirely on observational data, not experiments, and typically in response to unplanned fires where severity of impacts is interpreted to estimate fire intensity (Smith et al. 2016a, b). Our data clearly show that saplings of *P. engelmannii* and *T. plicata* do not match these expectations.

### Predicting fire-induced mortality

Comparison of crown scorch across fire intensities (Figs. 3, 4C, 5C) with 28-week mortality outcomes (Fig. 6) indicates that visual crown damage assessments are poor predictors of mid-term fire-induced sapling mortality, which likely explains the underperformance of these predictive models. However, excluding *Pinus palustris,* the dose–response relationships among the remaining seven species show relatively low variability and can be approximated by a single predictive model (Fig. 6C: Pm = 88.971 × FRE, r^2^ = 0.89, n = 37):

5 $${P}_{M(general)}=-0.893+108.55 x \frac{{FRE}^{3.93}}{{0.61}^{3.93}+{FRE}^{3.93}}$$

where, the residual standard error = 18.3, N = 37, Akaike Information Criterion (AIC) = 326 (as compared to a linear model of AIC = 332).

### Chlorophyll fluorescence trajectories as potential early-warning indicators

Post-fire Fv/Fm trajectories observed at dose levels > 0.6 MJ m⁻^2^ build on prior findings of short-term recovery followed by rapid decline and delayed mortality. Smith et al. (2017) observed a similar pattern in *Pinus contorta* var. *latifolia*, and Sparks et al. (2023) reported short-term post-fire Fv/Fm recovery in *Pinus monticola* and *Pseudotsuga menziesii*. Notably, the timing of the temporary recovery differs by species: 7–14 days post-fire in *Pinus contorta* var. *latifolia* (Smith et al. 2017), and 28- and 56-days post-fire in the current study for the 0.6 MJ m⁻^2^ and > 0.8 MJ m⁻^2^ treatments, respectively. This delayed temporary recovery response has now been observed with fire experiments in multiple conifer species, and it may be due to a delayed physiological or molecular stress response. Given that this temporary recovery appears to be both dose- and species-dependent, further research is needed to determine its causal physiological or molecular mechanisms and evaluate its potential as a prognostic indicator of fire-induced mortality in conifers. Repeated observations of chlorophyll fluorescence may provide an early warning indicator of tree death. For example, Sparks et al. (2024) showed that negative postfire chlorophyll fluorescence trajectories derived from repeated observations served as a reliable early warning sign of impending tree death in burned *Pinus monticola* and *Pseudotsuga menziesii* saplings.

### Future research to inform ecological management

Although across seven species, broadly similar relationships between FRE and probability of mortality were observed (Fig. 6C), each of these studies were conducted in the northwestern United States. This raises the question: Are the observed responses due to phenotypic plasticity to regional environmental conditions rather than evolutionary fire adaptations? Future research should replicate these experiments using potted nursery trials (to assess mortality) or common garden experiment provenance trials (to assess long-term recovery) with seed sources collected across the species’ native ranges to capture environmental variation. In the western United States, such experiments could focus on *Pinus ponderosa* or *Pseudotsuga menziesii* given their broad biological range and importance to forestry. Complementary research should also evaluate fire severity responses during landscape-scale wildfires, focusing on how plasticity varies across species and age classes in natural settings. Future research should also focus on conducting similar dose–response studies on older trees to assess how these relationships change as trees exhibit greater morphological and physiological protections to fire. Given the clear difference between the observed mortality in these young trees and the existing predictive models, more effort should be placed on repeating these dose–response experiments in natural fire setting to enable inclusion of relationships within predictive modeling systems.

In terms of the potential of chlorophyll fluorescence trajectories as potential early-warning indicators, we hypothesize that in these species, the trees are making a ‘last ditch’ effort to use available carbon stores to attempt to generate new tissue for fixing carbon, but cannot due to irrevocable damage to the cambium and phloem. We posit that damage to the cambium and phloem in these fire intensity doses are sufficiently catastrophic that the cambium is unable to repair the phloem and that non-structural carbohydrates (NSCs) resources above that height are used by the tree to enact the new tissues associated with the chlorophyll fluorescence response. We further posit in species that resprout that a similar response would be observed but through the production of new sprouts created through NSCs below the damaged area travelling down, where such species may be able to recover if the new growth is from belowground resources. This hypothesis could be assessed through a temporally intensive NSC study to assess how the NSCs fluctuate across the period of pre-, during- and the post-fire temporary recovery. Given related measures to Fv/Fm are obtainable at large spatial scales from solar-induced chlorophyll fluorescence (Mohammed et al. 2019; Tao et al. 2024), future studies could assess the utility of similar trajectories as potential early-warning indicators in mature trees across landscape scales.

## Conclusions

This study advances our understanding of how fire impacts conifers by quantifying the impact of increasing fire intensity on both mortality rates and physiological responses in *P. engelmannii* and *T. plicata* saplings. A clear dose–response relationship is shown with mortality rates increasing across fire intensity treatments. Physiological responses in Fv/Fm and crown scorch varied with FRE dose over time and may serve as useful indicators for predicting post-fire sapling mortality. Beyond insights into the fire physiology of *P. engelmannii*, these results can inform the design of future fire ecology experiments. Future research should examine genetic and physiological similarities among these species to identify phenotypic or molecular traits that could improve predictions of fire-induced mortality in conifers. Research should also evaluate whether these findings apply to naturally grown saplings and to older trees. Although rare, experiments involving mature trees could significantly improve our mechanistic understanding of species-specific fire responses (Sparks et al. 2017; Reed and Hood 2024; Smith et al. 2025).

## Supplementary Information

Supplementary Material 1: Table S1. Pre-fire and 1-week post-fire morphology and physiology measurements of *T. plicata* saplings under differing levels of fire intensity. Table S2. 2-week and 3-week post-fire morphology and physiology measurements of *T. plicata* saplings under differing levels of fire intensity. Table S3. 4-week and 8-week post-fire morphology and physiology measurements of *T. plicata* saplings under differing levels of fire intensity. Table S4. 16-week and 20-week post-fire morphology and physiology measurements of *T. plicata* saplings under differing levels of fire intensity. Table S5. 24-week and 28-week post-fire morphology and physiology measurements of *T. plicata* saplings under differing levels of fire intensity. Table S6. Pre-fire and 1-week post-fire morphology and physiology measurements of *P. engelmannii* saplings under differing levels of fire intensity. Table S7. 2-week and 3-week post-fire morphology and physiology measurements of *P. engelmannii* saplings under differing levels of fire intensity. Table S8. 4-week and 8-week post-fire morphology and physiology measurements of *P. engelmannii* saplings under differing levels of fire intensity Table S9. 16-week and 20-week post-fire morphology and physiology measurements of *P. engelmannii* saplings under differing levels of fire intensity. Table S10. 24-week and 28-week post-fire morphology and physiology measurements of *P. engelmannii* saplings under differing levels of fire intensity.

## Data Availability

All data are available via supplementary tables.

## Abbreviations

AIC: Akaike Information Criterion
BT: Bark thickness
DRC: Diameter at root collar
FOFEM: First Order Fire Effects Model
FRE: Fire Radiative Energy
Fv/Fm: Chlorophyll fluorescence
IFIRE: Idaho fire initiative for research and education
LAOC: *Larix occidentalis*
LD50: Lethal dose to cause 50% mortality
LFMC: Live fuel moisture content
LOAEL: Lowest-observed-adverse-effect-level
NOAEL: No-observed-adverse-effect-level
PCG: Percentage crown that is green
PIEN: *Picea Engelmannii*
PICO: *Pinus contorta.* Var. *latifolia*
PIMO: *Pinus monticola*
PIPO: *Pinus ponderosa*
PSME: *Pseudotsuga menziesii*
THPL: *Thuja plicata*
USDA: United States Department of Agriculture
