# Supplementary material for: Assessing the potential fire tolerance of conifer saplings in cold and wet environments using a pyro-ecophysiology approach
Source: Fire Ecol. 2026 Jan 13;22(1):14. doi: 10.1186/s42408-025-00443-7 (PMC12894159; doi:10.1186/s42408-025-00443-7)
Supplement: Supplementary file 1 — Supplementary Material 1: Table S1. Pre-fire and 1-week post-fire morphology and physiology measurements of T. plicata saplings under differing levels of fire intensity. Table S2. 2-week and 3-week post-fire morphology and physiology measurements of T. plicata saplings under differing levels of fire intensity. Table S3. 4-week and 8-week post-fire morphology and physiology measurements of T. plicata saplings under differing levels of fire intensity. Table S4. 16-week and 20-week post-fire morphology and physiology measurements of T. plicata saplings under differing levels of fire intensity. Table S5. 24-week and 28-week post-fire morphology and physiology measurements of T. plicata saplings under differing levels of fire intensity. Table S6. Pre-fire and 1-week post-fire morphology and physiology measurements of P. engelmannii saplings under differing levels of fire intensity. Table S7. 2-week and 3-week post-fire morphology and physiology measurements of P. engelmannii saplings under differing levels of fire intensity. Table S8. 4-week and 8-week post-fire morphology and physiology measurements of P. engelmannii saplings under differing levels of fire intensity Table S9. 16-week and 20-week post-fire morphology and physiology measurements of P. engelmannii saplings under differing levels of fire intensity. Table S10. 24-week and 28-week post-fire morphology and physiology measurements of P. engelmannii saplings under differing levels of fire intensity. [file 42408_2025_443_MOESM1_ESM.docx]

DOI TBD

Fire Ecology

**Supplementary Material**

**Assessing the potential fire tolerance of conifer saplings in cold and wet environments using a pyro-ecophysiology approach**

Alexander S. Blanco^1+^, David R. Wilson^2+^, Scott W. Rainsford^1+^, Grant L. Harley^1+^, Roshan P. Bhatta^+^, Corbin W. Halsey^1^, Gabriella M. Eldridge^1,3^, Daisy P. Estrada Garza^1^, L. May Brown^1^, Madeleine F. Stanley^1^, Jeffrey A. Logan^1^, Aaron M. Sparks^4^, Henry D. Adams^5^, Daniel M. Johnson^6^, Andrew T. Hudak^7^, Li Huang^1^, and Alistair M.S. Smith^1+*^

^1^ Department of Earth and Spatial Sciences, College of Science, University of Idaho, Moscow, ID, 83844, USA, blan1430@vandals.uidaho.edu, camp6674@vandals.uidaho.edu, brow9034@vandals.uidaho.edu, stan8938@vandals.uidaho.edu, gharley@uidaho.edu, rbhatta@uidaho.edu, hobbs95pine@gmail.com, lhuang@uidaho.edu, jeffreylogan@uidaho.edu, alistair@uidaho.edu (ORCID: 0000-0003-0071-9958),

^2^ Department of Civil and Environmental Engineering, College of Engineering, University of Idaho, Moscow, ID, 83844, USA, nemepotato@gmail.com

^4^ Department of Geography, Florida State University, Tallahassee, FL, 32306, USA, ge25@fsu.edu

^4^ Northwest Management Inc., Moscow, ID 83844, USA, asparks@northwestmanagement.com (ORCID: 0000-0003-1286-3770)

^5^ School of the Environment, Washington State University, Pullman, WA, 99164, USA, Henry.Adams@wsu.edu (ORCID: 0000-0001-9630-4305)

^6^ Warnell School of Forestry and Natural Resources, University of Georgia, Athens, GA, 30602, USA

danjohnson@uga.edu (ORCID: 0000-0003-1015-9560)

^7^ Rocky Mountain Research Station, United States Forest Service, Moscow, Idaho, 83844, USA, andrew.hudak@usda.gov

+ Equal contribution to paper

*Corresponding author: [alistair@uidaho.edu](mailto:alistair@uidaho.edu)

**Table S1. Pre-fire and 1-week post-fire morphology and physiology measurements of *Thuja plicata* saplings under differing levels of fire intensity.** ID refers to sapling tag number, FRE denotes the fire radiative energy dose (MJ m^-2^), height (cm), PCG denotes the percentage canopy that is green (%), DRC denotes diameter at root collar (mm), and Fv/Fm the chlorophyll fluorescence.

| Pre-fire data | | | | | | 1-week post-fire data | | | | | |
| --- | --- | --- | --- | --- | --- | --- | --- | --- | --- | --- | --- |
| ID | FRE | PCG | Height | DRC | Fv/FM | ID | FRE | PCG | Height | DRC | Fv/FM |
| 101 | - | - | 104.3 | 11.015 | 0.826 | 101 | 0.4 | 20 | 104.4 | 11.395 | 0.564 |
| 102 | - | - | 94.7 | 10.87 | 0.784 | 102 | 0.4 | 20 | 94.9 | 11.35 | 0.728 |
| 103 | - | - | 101.2 | 13.15 | 0.819 | 103 | 0.4 | 25 | 101.3 | 13.16 | 0.272 |
| 104 | - | - | 97.6 | 12.535 | 0.834 | 104 | 0.4 | 30 | 99.5 | 13.055 | 0.616 |
| 105 | - | - | 98.8 | 11.64 | 0.827 | 105 | 0.4 | 15 | 98.9 | 12.61 | 0.466 |
| 106 | - | - | 108.6 | 13.575 | 0.822 | 106 | 0.4 | 20 | 108.4 | 13.23 | 0.764 |
| 107 | - | - | 116.4 | 12.575 | 0.811 | 107 | 0.4 | 25 | 1178 | 12.68 | 0.635 |
| 108 | - | - | 103.9 | 12.78 | 0.814 | 108 | 0.6 | 15 | 104.3 | 13 | 0.569 |
| 109 | - | - | 112.2 | 10.42 | 0.82 | 109 | 0.6 | 15 | 114.2 | 10.435 | 0.665 |
| 110 | - | - | 109.3 | 12.72 | 0.79 | 110 | 0.6 | 5 | 109.2 | 12.865 | 0.176 |
| 111 | - | - | 103.2 | 12.815 | 0.794 | 111 | 0.6 | 20 | 102.4 | 12.955 | 0.782 |
| 112 | - | - | 93.7 | 9.915 | 0.819 | 112 | 0.6 | 5 | 94.5 | 11.045 | 0.009 |
| 113 | - | - | 99.6 | 11.535 | 0.788 | 113 | 0.6 | 10 | 99.7 | 11.18 | 0.006 |
| 114 | - | - | 103.1 | 10.67 | 0.847 | 114 | 0.6 | 10 | 103.6 | 10.84 | 0.094 |
| 115 | - | - | 108.9 | 12.2 | 0.835 | 115 | 0.8 | 5 | 108.8 | 12.755 | 0.008 |
| 116 | - | - | 106.9 | 12.535 | 0.822 | 116 | 0.8 | 0 | 107.6 | 12.765 | 0.021 |
| 117 | - | - | 96.7 | 12.325 | 0.814 | 117 | 0.8 | 5 | 97.6 | 13.03 | 0.006 |
| 118 | - | - | 106.6 | 11.155 | 0.799 | 118 | 0.8 | 0 | 106.1 | 12.275 | 0.002 |
| 119 | - | - | 92.6 | 10.945 | 0.815 | 119 | 0.8 | 10 | 92.3 | 10.995 | 0.787 |
| 120 | - | - | 107.1 | 12.53 | 0.808 | 120 | 0.8 | 2 | 108.4 | 12.635 | 0.02 |
| 121 | - | - | 91.6 | 10.385 | 0.817 | 121 | 0.8 | 0 | 92.2 | 10.525 | 0.002 |
| 122 | - | - | 107.7 | 12.175 | 0.814 | 122 | 0.2 | 55 | 107.9 | 12.445 | 0.743 |
| 123 | - | - | 104.7 | 11.57 | 0.786 | 123 | 0.2 | 50 | 104.4 | 12.13 | 0.786 |
| 124 | - | - | 109.7 | 12.685 | 0.797 | 124 | 0.2 | 45 | 109.5 | 12.16 | 0.784 |
| 125 | - | - | 92.9 | 11.82 | 0.818 | 125 | 0.2 | 70 | 94.1 | 11.73 | 0.809 |
| 126 | - | - | 94.8 | 9.13 | 0.821 | 126 | 0.2 | 70 | 97.1 | 9.32 | 0.819 |
| 127 | - | - | 99.7 | 12.21 | 0.842 | 127 | 0.2 | 60 | 99.6 | 13.575 | 0.823 |
| 128 | - | - | 115.4 | 12.8 | 0.782 | 128 | 0.2 | 65 | 113.8 | 13.01 | 0.807 |
| 129 | - | - | 94.1 | 11.635 | 0.802 | 129 | 1.0 | 0 | 93.8 | 12.12 | 0.016 |
| 130 | - | - | 108.6 | 12.475 | 0.793 | 130 | 1.0 | 1 | 110.8 | 12.765 | 0.021 |
| 131 | - | - | 105.3 | 13.495 | 0.814 | 131 | 1.0 | 5 | 105.3 | 13.16 | 0.003 |
| 132 | - | - | 107.1 | 12.95 | 0.754 | 132 | 1.0 | 2 | 107.2 | 13.4 | 0.008 |
| 133 | - | - | 107.4 | 11.41 | 0.83 | 133 | 1.0 | 1 | 110.3 | 12.415 | 0.005 |
| 134 | - | - | 98.7 | 10.495 | 0.803 | 134 | 1.0 | 1 | 102.3 | 10.565 | 0.01 |
| 135 | - | - | 105.9 | 10.01 | 0.819 | 135 | 1.0 | 2 | 109.6 | 9.96 | 0.021 |
| 136 | - | - | 112.1 | 17.165 | 0.787 | 136 | 0 | 95 | 114.2 | 20.445 | 0.794 |
| 137 | - | - | 105.5 | 11.455 | 0.801 | 137 | 0 | 95 | 106.1 | 12.51 | 0.82 |
| 138 | - | - | 108.2 | 13.015 | 0.813 | 138 | 0 | 100 | 110.1 | 15.045 | 0.751 |
| 139 | - | - | 99.6 | 10.74 | 0.814 | 139 | 0 | 100 | 103.2 | 11.13 | 0.85 |
| 140 | - | - | 101.8 | 11.46 | 0.826 | 140 | 0 | 100 | 102.8 | 11.92 | 0.792 |
| 141 | - | - | 105.4 | 13.755 | 0.816 | 141 | 0 | 100 | 107.6 | 15.105 | 0.807 |
| 142 | - | - | 103.2 | 12.145 | 0.795 | 142 | 0 | 95 | 105.2 | 12.34 | 0.774 |

**Table S2. 2-week and 3-week post-fire morphology and physiology measurements of *Thuja plicata* saplings under differing levels of fire intensity.** ID refers to sapling tag number, FRE denotes the fire radiative energy dose (MJ m^-2^), height (cm), PCG denotes the percentage canopy that is green (%), DRC denotes diameter at root collar (mm), and Fv/Fm the chlorophyll fluorescence.

| 2-week post-fire data | | | | | | 3-week post-fire data | | | | | |
| --- | --- | --- | --- | --- | --- | --- | --- | --- | --- | --- | --- |
| ID | FRE | PCG | Height | DRC | Fv/FM | ID | FRE | PCG | Height | DRC | Fv/FM |
| ID | FRE | 20 | 105.5 | 11.12 | 0.691 | ID | FRE | 15 | 105.9 | 11.84 | 0.789 |
| 101 | 0.4 | 15 | 94.5 | 10.645 | 0.664 | 101 | 0.4 | 15 | 94.9 | 10.605 | 0.742 |
| 102 | 0.4 | 20 | 101 | 12.715 | 0.564 | 102 | 0.4 | 15 | 102.6 | 13.79 | 0.728 |
| 103 | 0.4 | 30 | 98.5 | 13.22 | 0.584 | 103 | 0.4 | 25 | 99.4 | 13.265 | 0.81 |
| 104 | 0.4 | 15 | 99.5 | 11.45 | 0.413 | 104 | 0.4 | 10 | 99.8 | 12.27 | 0.534 |
| 105 | 0.4 | 35 | 109.5 | 13.545 | 0.781 | 105 | 0.4 | 25 | 109.6 | 13.565 | 0.776 |
| 106 | 0.4 | 25 | 118.2 | 12.325 | 0.708 | 106 | 0.4 | 30 | 119.1 | 13 | 0.827 |
| 107 | 0.4 | 20 | 104 | 12.71 | 0.760 | 107 | 0.4 | 20 | 104.9 | 13.5 | 0.666 |
| 108 | 0.6 | 25 | 115.4 | 10.395 | 0.740 | 108 | 0.6 | 20 | 115 | 10.5 | 0.787 |
| 109 | 0.6 | 10 | 108.9 | 13.08 | 0.663 | 109 | 0.6 | 10 | 110.3 | 13.28 | 0.408 |
| 110 | 0.6 | 25 | 104.6 | 12.955 | 0.744 | 110 | 0.6 | 20 | 105.3 | 12.97 | 0.761 |
| 111 | 0.6 | 5 | 95.5 | 9.915 | 0.014 | 111 | 0.6 | 5 | 90.3 | 11.06 | 0.012 |
| 112 | 0.6 | 10 | 99.8 | 11.685 | 0.093 | 112 | 0.6 | 15 | 99.1 | 11.775 | 0.097 |
| 113 | 0.6 | 10 | 104 | 10.625 | 0.497 | 113 | 0.6 | 5 | 104.3 | 11.06 | 0.016 |
| 114 | 0.6 | 2 | 109.7 | 12.05 | 0.016 | 114 | 0.6 | 1 | 109.9 | 12.4 | 0.035 |
| 115 | 0.8 | 0 | 109.4 | 12.975 | 0.092 | 115 | 0.8 | 0 | 108.4 | 13.435 | 0.046 |
| 116 | 0.8 | 2 | 98.7 | 12.22 | 0.000 | 116 | 0.8 | 1 | 97.8 | 13.17 | 0.074 |
| 117 | 0.8 | 0 | 108.5 | 12.18 | 0.020 | 117 | 0.8 | 0 | 106.8 | 12.075 | 0.034 |
| 118 | 0.8 | 10 | 94.5 | 10.51 | 0.692 | 118 | 0.8 | 10 | 95.4 | 12.03 | 0.443 |
| 119 | 0.8 | 0 | 110.2 | 11.915 | 0.032 | 119 | 0.8 | 0 | 108.3 | 14.025 | 0.066 |
| 120 | 0.8 | 0 | 93.5 | 10.035 | 0.011 | 120 | 0.8 | 0 | 93.3 | 10.205 | 0.005 |
| 121 | 0.8 | 45 | 109.7 | 11.815 | 0.752 | 121 | 0.8 | 50 | 107.6 | 12.875 | 0.821 |
| 122 | 0.2 | 50 | 105.5 | 11.78 | 0.788 | 122 | 0.2 | 50 | 105.1 | 11.705 | 0.808 |
| 123 | 0.2 | 60 | 111.8 | 11.91 | 0.766 | 123 | 0.2 | 65 | 110.6 | 12.685 | 0.792 |
| 124 | 0.2 | 80 | 95 | 11.89 | 0.813 | 124 | 0.2 | 80 | 94.2 | 11.84 | 0.823 |
| 125 | 0.2 | 70 | 98 | 9.295 | 0.811 | 125 | 0.2 | 90 | 97.8 | 9.245 | 0.838 |
| 126 | 0.2 | 65 | 101.7 | 12.33 | 0.780 | 126 | 0.2 | 75 | 102.6 | 13.89 | 0.827 |
| 127 | 0.2 | 90 | 116.8 | 12.345 | 0.766 | 127 | 0.2 | 85 | 116.7 | 712.38 | 0.8 |
| 128 | 0.2 | 0 | 94.9 | 12.08 | 0.033 | 128 | 0.2 | 0 | 94.6 | 13.31 | 0.035 |
| 129 | 1.0 | 1 | 111.2 | 12.475 | 0.086 | 129 | 1.0 | 1 | 112 | 13.1 | 0.041 |
| 130 | 1.0 | 5 | 106.8 | 12.89 | 0.184 | 130 | 1.0 | 5 | 106.6 | 13.27 | 0.058 |
| 131 | 1.0 | 2 | 107.2 | 12.475 | 0.006 | 131 | 1.0 | 1 | 107.4 | 13.06 | 0.01 |
| 132 | 1.0 | 1 | 110 | 10.79 | 0.000 | 132 | 1.0 | 0 | 110.9 | 12.105 | 0.059 |
| 133 | 1.0 | 1 | 103 | 11.015 | 0.000 | 133 | 1.0 | 1 | 101.6 | 9.915 | 0.063 |
| 134 | 1.0 | 2 | 110.8 | 9.76 | 0.000 | 134 | 1.0 | 1 | 109.8 | 10.135 | 0.037 |
| 135 | 1.0 | 95 | 116 | 17.315 | 0.789 | 135 | 1.0 | 95 | 1151 | 17.185 | 0.815 |
| 136 | 0 | 99 | 107.8 | 11.035 | 0.790 | 136 | 0 | 100 | 107.1 | 12.13 | 0.811 |
| 137 | 0 | 100 | 109.9 | 14.005 | 0.792 | 137 | 0 | 100 | 110.6 | 15.345 | 0.835 |
| 138 | 0 | 100 | 104.3 | 11.715 | 0.836 | 138 | 0 | 100 | 103.8 | 11.12 | 0.824 |
| 139 | 0 | 99 | 104.2 | 11.71 | 0.831 | 139 | 0 | 99 | 104.2 | 11.84 | 0.829 |
| 140 | 0 | 100 | 107.3 | 16.385 | 0.785 | 140 | 0 | 100 | 108.9 | 15.16 | 0.786 |
| 141 | 0 | 99 | 107.2 | 12.555 | 0.806 | 141 | 0 | 99 | 107 | 12.525 | 0.81 |

**Table S3. 4-week and 8-week post-fire morphology and physiology measurements of *Thuja plicata* saplings under differing levels of fire intensity.** ID refers to sapling tag number, FRE denotes the fire radiative energy dose (MJ m^-2^), height (cm), PCG denotes the percentage canopy that is green (%), DRC denotes diameter at root collar (mm), and Fv/Fm the chlorophyll fluorescence.

| 4-week post-fire data | | | | | | 8-week post-fire data | | | | | |
| --- | --- | --- | --- | --- | --- | --- | --- | --- | --- | --- | --- |
| ID | FRE | PCG | Height | DRC | Fv/FM | ID | FRE | PCG | Height | DRC | Fv/FM |
| 101 | 0.4 | 15 | 105.3 | 5.905 | 0.724 | ID | FRE | 15 | 105.2 | 11.38 | 0.746 |
| 102 | 0.4 | 15 | 95.4 | 10.305 | 0.685 | 101 | 0.4 | 15 | 95.3 | 11.31 | 0.269 |
| 103 | 0.4 | 15 | 101.9 | 13.25 | 0.692 | 102 | 0.4 | 15 | 101.2 | 13.725 | 0.662 |
| 104 | 0.4 | 20 | 99.9 | 12.89 | 0.786 | 103 | 0.4 | 20 | 99.7 | 13.095 | 0.832 |
| 105 | 0.4 | 10 | 97.8 | 12.05 | 0.698 | 104 | 0.4 | 10 | 99.3 | 12.37 | 0.642 |
| 106 | 0.4 | 25 | 107.8 | 13.13 | 0.675 | 105 | 0.4 | 25 | 107.4 | 14.2 | 0.809 |
| 107 | 0.4 | 25 | 117.8 | 12.495 | 0.805 | 106 | 0.4 | 25 | 118.4 | 13.7 | 0.833 |
| 108 | 0.6 | 10 | 105.9 | 13.25 | 0.706 | 107 | 0.4 | 20 | 106.6 | 13.8 | 0.804 |
| 109 | 0.6 | 20 | 114.2 | 10.515 | 0.797 | 108 | 0.6 | 15 | 112.2 | 10.36 | 0.816 |
| 110 | 0.6 | 5 | 108.6 | 12.32 | 0.731 | 109 | 0.6 | 5 | 110.3 | 13.05 | 0.778 |
| 111 | 0.6 | 20 | 104.7 | 13.1 | 0.759 | 110 | 0.6 | 15 | 105.1 | 12.755 | 0.652 |
| 112 | 0.6 | 5 | 94.3 | 10.955 | 0.228 | 111 | 0.6 | 5 | 93.1 | 10.705 | 0.079 |
| 113 | 0.6 | 10 | 99.4 | 11.245 | 0.738 | 112 | 0.6 | 5 | 100.2 | 602.715 | 0.611 |
| 114 | 0.6 | 10 | 102.9 | 10.71 | 0.594 | 113 | 0.6 | 5 | 103.9 | 11.05 | 0.697 |
| 115 | 0.8 | 1 | 109.4 | 12.645 | 0.05 | 114 | 0.6 | 0 | 109.4 | 12.81 | 0.094 |
| 116 | 0.8 | 0 | 108.7 | 12.285 | 0.09 | 115 | 0.8 | 0 | 107.8 | 14.475 | 0.043 |
| 117 | 0.8 | 1 | 98.4 | 12.395 | 0.038 | 116 | 0.8 | 0 | 97.8 | 13.76 | 0.119 |
| 118 | 0.8 | 0 | 106.4 | 12.05 | 0.104 | 117 | 0.8 | 0 | 106.1 | 12.27 | 0.000 |
| 119 | 0.8 | 10 | 93.6 | 11.95 | 0.102 | 118 | 0.8 | 5 | 94.2 | 12.22 | 0.100 |
| 120 | 0.8 | 0 | 107.4 | 13.88 | 0.101 | 119 | 0.8 | 0 | 107.5 | 13.12 | 0.063 |
| 121 | 0.8 | 0 | 92.8 | 10.725 | 0.023 | 120 | 0.8 | 0 | 93.3 | 10.145 | 0.018 |
| 122 | 0.2 | 45 | 110.6 | 12.965 | 0.803 | 121 | 0.8 | 50 | 108.1 | 13.7 | 0.820 |
| 123 | 0.2 | 60 | 106.6 | 11.28 | 0.782 | 122 | 0.2 | 45 | 105.4 | 12.22 | 0.819 |
| 124 | 0.2 | 50 | 111.2 | 12.36 | 0.774 | 123 | 0.2 | 55 | 108.9 | 13.54 | 0.755 |
| 125 | 0.2 | 70 | 93.8 | 11.805 | 0.819 | 124 | 0.2 | 65 | 95.3 | 11.745 | 0.837 |
| 126 | 0.2 | 65 | 97.8 | 9.205 | 0.812 | 125 | 0.2 | 70 | 97.4 | 9.955 | 0.836 |
| 127 | 0.2 | 60 | 101.2 | 13.435 | 0.817 | 126 | 0.2 | 65 | 101.9 | 13.99 | 0.829 |
| 128 | 0.2 | 80 | 115.9 | 12.535 | 0.772 | 127 | 0.2 | 75 | 114.4 | 12.67 | 0.755 |
| 129 | 1.0 | 0 | 93.1 | 12.835 | 0.037 | 128 | 0.2 | 0 | 93.1 | 13.07 | 0.049 |
| 130 | 1.0 | 0 | 110.2 | 13.49 | 0.087 | 129 | 1.0 | 2 | 109.6 | 13.41 | 0.373 |
| 131 | 1.0 | 5 | 105.8 | 12.925 | 0.027 | 130 | 1.0 | 2 | 106.4 | 13.49 | 0.718 |
| 132 | 1.0 | 5 | 108.1 | 12.135 | 0.025 | 131 | 1.0 | 2 | 107.1 | 14.01 | 0.255 |
| 133 | 1.0 | 0 | 109.1 | 11.21 | 0.181 | 132 | 1.0 | 0 | 109.4 | 12.445 | 0.048 |
| 134 | 1.0 | 1 | 100.1 | 9.6 | 0.02 | 133 | 1.0 | 0 | 101.1 | 10.23 | 0.068 |
| 135 | 1.0 | 2 | 106.5 | 9.765 | 0.121 | 134 | 1.0 | 2 | 107.4 | 10.15 | 0.030 |
| 136 | 0 | 95 | 115.2 | 16.8 | 0.788 | 135 | 1.0 | 100 | 114.6 | 18.845 | 0.755 |
| 137 | 0 | 100 | 105.4 | 11.06 | 0.799 | 136 | 0 | 100 | 105.7 | 12.85 | 0.824 |
| 138 | 0 | 100 | 109.6 | 14.7 | 0.794 | 137 | 0 | 100 | 108.9 | 14.125 | 0.826 |
| 139 | 0 | 100 | 103.5 | 11.085 | 0.747 | 138 | 0 | 100 | 103.9 | 11.295 | 0.810 |
| 140 | 0 | 100 | 101.3 | 11.54 | 0.797 | 139 | 0 | 100 | 103.7 | 11.935 | 0.820 |
| 141 | 0 | 100 | 108.3 | 14.34 | 0.75 | 140 | 0 | 100 | 108.7 | 14.79 | 0.822 |
| 142 | 0 | 95 | 106.3 | 13.215 | 0.814 | 141 | 0 | 90 | 106.4 | 12.36 | 0.796 |

**Table S4. 16-week and 20-week post-fire morphology and physiology measurements of *Thuja plicata* saplings under differing levels of fire intensity.** ID refers to sapling tag number, FRE denotes the fire radiative energy dose (MJ m^-2^), height (cm), PCG denotes the percentage canopy that is green (%), DRC denotes diameter at root collar (mm), and Fv/Fm the chlorophyll fluorescence.

| 16-week post-fire data | | | | | | 20-week post-fire data | | | | | |
| --- | --- | --- | --- | --- | --- | --- | --- | --- | --- | --- | --- |
| ID | FRE | PCG | Height | DRC | Fv/FM | ID | FRE | PCG | Height | DRC | Fv/FM |
| 101 | 0.4 | 15 | 105.1 | 11.21 | 0.11 | ID | FRE | 0 | 105.1 | 11.845 | 0.009 |
| 102 | 0.4 | 15 | 95.4 | 10.71 | 0.688 | 101 | 0.4 | 15 | 95.2 | 11.965 | .684/ |
| 103 | 0.4 | 15 | 101.7 | 14.165 | 0.723 | 102 | 0.4 | 15 | 100.8 | 13.27 | .428/ |
| 104 | 0.4 | 25 | 99.8 | 13.425 | 0.772 | 103 | 0.4 | 25 | 99.2 | 13.935 | 0.814 |
| 105 | 0.4 | 10 | 99.4 | 12.54 | 0.706 | 104 | 0.4 | 10 | 99.8 | 12.5 | 0.779 |
| 106 | 0.4 | 30 | 108.1 | 14.06 | 0.794 | 105 | 0.4 | 25 | 109.8 | 14.225 | 0.824 |
| 107 | 0.4 | 25 | 116.3 | 13.725 | 0.742 | 106 | 0.4 | 30 | 118.4 | 13.415 | 0.805 |
| 108 | 0.6 | 15 | 104.2 | 13.295 | 0.209 | 107 | 0.4 | 5 | 104.6 | 13.49 | 0.006 |
| 109 | 0.6 | 20 | 112.5 | 10.455 | 0.75 | 108 | 0.6 | 20 | 114.7 | 10.53 | 0.76 |
| 110 | 0.6 | 10 | 108.9 | 13.125 | 0.444 | 109 | 0.6 | 5 | 109.6 | 13.25 | 0.693 |
| 111 | 0.6 | 15 | 105.3 | 13.615 | 0.479 | 110 | 0.6 | 15 | 104.3 | 12.825 | 0 |
| 112 | 0.6 | 2 | 91.9 | 10.81 | 0.53 | 111 | 0.6 | 1 | 90.6 | 10.91 | 0.153 |
| 113 | 0.6 | 15 | 98.2 | 12.265 | 0.451 | 112 | 0.6 | 1 | 97.9 | 13.2 | 0.471 |
| 114 | 0.6 | 5 | 103.4 | 11.385 | 0.573 | 113 | 0.6 | 1 | 100.2 | 10.815 | 0.011 |
| 115 | 0.8 | 0 | 106.9 | 12.385 | 0 | 114 | 0.6 | 0 | 107.2 | 13.845 | 0 |
| 116 | 0.8 | 0 | 108.1 | 14.13 | 0 | 115 | 0.8 | 0 | 106.8 | 13 | 0 |
| 117 | 0.8 | 0 | 97.3 | 13.3 | 0 | 116 | 0.8 | 0 | 97.4 | 13.205 | 0 |
| 118 | 0.8 | 0 | 105.4 | 11.635 | 0 | 117 | 0.8 | 0 | 104.3 | 12.445 | 0 |
| 119 | 0.8 | 5 | 94.1 | 11.645 | 0 | 118 | 0.8 | 0 | 93.7 | 11.21 | 0.145 |
| 120 | 0.8 | 0 | 107.2 | 13.44 | 0 | 119 | 0.8 | 0 | 106.6 | 13.585 | 0 |
| 121 | 0.8 | 0 | 91.6 | 11.17 | 0 | 120 | 0.8 | 0 | 92.7 | 10.145 | 0 |
| 122 | 0.2 | 50 | 108.6 | 12.93 | 0.783 | 121 | 0.8 | 50 | 108.1 | 13.315 | 0.793 |
| 123 | 0.2 | 50 | 104.7 | 11.97 | 0.724 | 122 | 0.2 | 55 | 104.9 | 14.135 | 0.785 |
| 124 | 0.2 | 50 | 110.7 | 13.605 | 0.67 | 123 | 0.2 | 50 | 108.7 | 13.45 | 0.695 |
| 125 | 0.2 | 65 | 94.2 | 12.005 | 0.788 | 124 | 0.2 | 75 | 94.3 | 13.24 | 0.801 |
| 126 | 0.2 | 65 | 97.6 | 9.895 | 0.802 | 125 | 0.2 | 80 | 97.1 | 10.85 | 0.812 |
| 127 | 0.2 | 60 | 99.8 | 14.34 | 0.773 | 126 | 0.2 | 65 | 101.7 | 14.825 | 0.788 |
| 128 | 0.2 | 80 | 113.4 | 13.53 | 0.714 | 127 | 0.2 | 90 | 115.1 | 13.49 | 0.769 |
| 129 | 1.0 | 0 | 93.1 | 13.12 | 0 | 128 | 0.2 | 0 | 92.1 | 14.125 | 0 |
| 130 | 1.0 | 0 | 110.2 | 13.915 | 0.179 | 129 | 1.0 | 0 | 107.8 | 14.3 | 0 |
| 131 | 1.0 | 5 | 105.1 | 13.58 | 0.008 | 130 | 1.0 | 0 | 105.6 | 13.745 | 0 |
| 132 | 1.0 | 1 | 107.6 | 14.21 | 0 | 131 | 1.0 | 0 | 107.4 | 13.005 | 0 |
| 133 | 1.0 | 0 | 105.7 | 11.235 | 0 | 132 | 1.0 | 0 | 109.9 | 12.445 | 0 |
| 134 | 1.0 | 0 | 99.3 | 10.525 | 0.037 | 133 | 1.0 | 0 | 100.2 | 10.18 | 0 |
| 135 | 1.0 | 0 | 106.1 | 10.5 | 0 | 134 | 1.0 | 0 | 105.9 | 10.76 | 0 |
| 136 | 0 | 95 | 114.2 | 18.315 | 0.728 | 135 | 1.0 | 100 | 113.5 | 18.025 | 0.732 |
| 137 | 0 | 100 | 104.5 | 12.24 | 0.738 | 136 | 0 | 100 | 107.6 | 13.98 | 0.738 |
| 138 | 0 | 100 | 108.6 | 15.18 | 0.784 | 137 | 0 | 100 | 109.4 | 15.45 | 0.782 |
| 139 | 0 | 100 | 101.9 | 11.3 | 0.744 | 138 | 0 | 100 | 102.6 | 12.26 | 0.749 |
| 140 | 0 | 100 | 101.6 | 12.765 | 0.753 | 139 | 0 | 100 | 103.2 | 12.635 | 0.767 |
| 141 | 0 | 100 | 108.7 | 16.7 | 0.69 | 140 | 0 | 100 | 108.4 | 14.765 | 0.729 |
| 142 | 0 | 50 | 106.4 | 12.68 | 0.737 | 141 | 0 | 40 | 106.8 | 13.275 | 0.767 |

**Table S5. 24-week and 28-week post-fire morphology and physiology measurements of *Thuja plicata* saplings under differing levels of fire intensity.** ID refers to sapling tag number, FRE denotes the fire radiative energy dose (MJ m^-2^), height (cm), PCG denotes the percentage canopy that is green (%), DRC denotes diameter at root collar (mm), and Fv/Fm the chlorophyll fluorescence.

| 24-week post-fire data | | | | | | 28-week post-fire data | | | |
| --- | --- | --- | --- | --- | --- | --- | --- | --- | --- |
| ID | FRE | PCG | Height | DRC | Fv/FM | ID | FRE | PCG | Status |
| 101 | 0.4 | 0 | 102.6 | 11.235 | 0 | ID | 0.4 | 20 | Dead |
| 102 | 0.4 | 10 | 94.5 | 10.96 | 0.791 | 101 | 0.4 | 20 | Alive |
| 103 | 0.4 | 10 | 102.1 | 14.035 | 0.714 | 102 | 0.4 | 25 | Alive |
| 104 | 0.4 | 20 | 100.4 | 13.705 | 0.794 | 103 | 0.4 | 30 | Alive |
| 105 | 0.4 | 10 | 106.8 | 12.365 | 0.785 | 104 | 0.4 | 15 | Alive |
| 106 | 0.4 | 25 | 109.1 | 15.065 | 0.731 | 105 | 0.4 | 20 | Alive |
| 107 | 0.4 | 30 | 118.6 | 14.115 | 0.807 | 106 | 0.4 | 25 | Alive |
| 108 | 0.6 | 0 | 103.4 | 13.175 | 0 | 107 | 0.6 | 15 | Dead |
| 109 | 0.6 | 20 | 112.6 | 10.465 | 0.703 | 108 | 0.6 | 15 | Alive |
| 110 | 0.6 | 1 | 108.7 | 13.345 | 0 | 109 | 0.6 | 5 | Dead |
| 111 | 0.6 | 0 | 101 | 12.815 | 0.015 | 110 | 0.6 | 20 | Dead |
| 112 | 0.6 | 0 | 91.1 | 10.47 | 0 | 111 | 0.6 | 5 | Dead |
| 113 | 0.6 | 0 | 98.7 | 12.62 | 0 | 112 | 0.6 | 10 | Dead* |
| 114 | 0.6 | 0 | 99.9 | 11.185 | 0 | 113 | 0.6 | 10 | Dead |
| 115 | 0.8 | 0 | 106.8 | 12.715 | 0 | 114 | 0.8 | 5 | Dead |
| 116 | 0.8 | 0 | 106.5 | 13.17 | 0 | 115 | 0.8 | 0 | Dead |
| 117 | 0.8 | 0 | 96.7 | 12.735 | 0 | 116 | 0.8 | 5 | Dead |
| 118 | 0.8 | 0 | 104.9 | 11.97 | 0 | 117 | 0.8 | 0 | Dead |
| 119 | 0.8 | 0 | 93.3 | 11.35 | 0 | 118 | 0.8 | 10 | Dead |
| 120 | 0.8 | 0 | 105.7 | 12.535 | 0 | 119 | 0.8 | 2 | Dead |
| 121 | 0.8 | 0 | 90.4 | 10.31 | 0 | 120 | 0.8 | 0 | Dead |
| 122 | 0.2 | 60 | 107.4 | 13.045 | 0.759 | 121 | 0.2 | 55 | Alive |
| 123 | 0.2 | 60 | 105.9 | 12.695 | 0.745 | 122 | 0.2 | 50 | Alive |
| 124 | 0.2 | 55 | 112.1 | 13.255 | 0.62 | 123 | 0.2 | 45 | Alive |
| 125 | 0.2 | 60 | 94.4 | 12.915 | 0.816 | 124 | 0.2 | 70 | Alive |
| 126 | 0.2 | 65 | 97.3 | 11.345 | 0.787 | 125 | 0.2 | 70 | Alive |
| 127 | 0.2 | 60 | 99.7 | 14.805 | 0.761 | 126 | 0.2 | 60 | Alive |
| 128 | 0.2 | 80 | 114.1 | 14.02 | 0.766 | 127 | 0.2 | 65 | Alive |
| 129 | 1.0 | 0 | 89.1 | 12.83 | 0 | 128 | 1.0 | 0 | Dead |
| 130 | 1.0 | 0 | 109.7 | 14.27 | 0 | 129 | 1.0 | 1 | Dead |
| 131 | 1.0 | 0 | 105.1 | 12.805 | 0 | 130 | 1.0 | 5 | Dead |
| 132 | 1.0 | 0 | 106.4 | 12.53 | 0 | 131 | 1.0 | 2 | Dead |
| 133 | 1.0 | 0 | 105.2 | 11.4 | 0 | 132 | 1.0 | 1 | Dead |
| 134 | 1.0 | 0 | 98.4 | 9.845 | 0 | 133 | 1.0 | 1 | Dead |
| 135 | 1.0 | 0 | 105.2 | 9.81 | 0 | 134 | 1.0 | 2 | Dead |
| 136 | 0 | 95 | 113.8 | 17.82 | 0.745 | 135 | 0 | 95 | Alive |
| 137 | 0 | 100 | 102.9 | 13.365 | 0.767 | 136 | 0 | 95 | Alive |
| 138 | 0 | 100 | 108.6 | 15.685 | 0.753 | 137 | 0 | 100 | Alive |
| 139 | 0 | 100 | 103.2 | 14.475 | 0.789 | 138 | 0 | 100 | Alive |
| 140 | 0 | 100 | 101.9 | 12.98 | 0.813 | 139 | 0 | 100 | Alive |
| 141 | 0 | 100 | 108.9 | 15.34 | 0.746 | 140 | 0 | 100 | Alive |
| 142 | 0 | 70 | 106.5 | 12.715 | 0.801 | 141 | 0 | 95 | Alive |

**Table S6. Pre-fire and 1-week post-fire morphology and physiology measurements of *Picea engelmannii* saplings under differing levels of fire intensity.** ID refers to sapling tag number, FRE denotes the fire radiative energy dose (MJ m^-2^), height (cm), PCG denotes the percentage canopy that is green (%), DRC denotes diameter at root collar (mm), and Fv/Fm the chlorophyll fluorescence.

| Pre-fire data | | | | | | 1-week post-fire data | | | | | |
| --- | --- | --- | --- | --- | --- | --- | --- | --- | --- | --- | --- |
| ID | FRE | PCG | Height | DRC | Fv/FM | ID | FRE | PCG | Height | DRC | Fv/FM |
| 701 | 0.4 | 100 | 60.3 | 9.415 | 0.806 | 701 | 0.4 | 40 | 57.8 | 9.65 | 0.72 |
| 702 | 0.4 | 100 | 50.7 | 10.46 | 0.814 | 702 | 0.4 | 35 | 50.2 | 10.21 | 0.662 |
| 703 | 0.4 | 100 | 47.8 | 10.14 | 0.849 | 703 | 0.4 | 30 | 48.3 | 9.88 | 0.551 |
| 704 | 0.4 | 100 | 53.6 | 10.57 | 0.848 | 704 | 0.4 | 30 | 53.6 | 9.96 | 0.772 |
| 705 | 0.4 | 100 | 53.6 | 9.575 | 0.81 | 705 | 0.4 | 35 | 54.9 | 9.75 | 0.757 |
| 706 | 0.4 | 100 | 59.5 | 9.775 | 0.839 | 706 | 0.4 | 40 | 59.9 | 9.77 | 0.577 |
| 707 | 0.4 | 100 | 54.6 | 11.985 | 0.837 | 707 | 0.4 | 45 | 55.4 | 11.88 | 0.847 |
| 708 | 0.6 | 100 | 50.9 | 8.945 | 0.826 | 708 | 0.6 | 10 | 51.2 | 9.44 | 0.754 |
| 709 | 0.6 | 100 | 55.1 | 9.78 | 0.809 | 709 | 0.6 | 5 | 55.1 | 10.56 | 0.013 |
| 710 | 0.6 | 100 | 51.6 | 12.12 | 0.832 | 710 | 0.6 | 10 | 50.9 | 13.86 | 0.685 |
| 711 | 0.6 | 100 | 57.7 | 12.895 | 0.813 | 711 | 0.6 | 15 | 57.2 | 12.92 | 0.628 |
| 712 | 0.6 | 100 | 56.4 | 10.28 | 0.832 | 712 | 0.6 | 10 | 57.7 | 10.56 | 0.27 |
| 713 | 0.6 | 100 | 67 | 10.07 | 0.819 | 713 | 0.6 | 5 | 66.5 | 11.09 | 0.677 |
| 714 | 0.6 | 100 | 61.9 | 10.78 | 0.782 | 714 | 0.6 | 1 | 62.9 | 10.27 | 0.31 |
| 715 | 0.8 | 100 | 59.3 | 12.375 | 0.831 | 715 | 0.8 | 5 | 60.1 | 12.18 | 0.162 |
| 716 | 0.8 | 100 | 64.4 | 12.665 | 0.833 | 716 | 0.8 | 10 | 64.4 | 11.63 | 0.694 |
| 717 | 0.8 | 100 | 66.2 | 12.815 | 0.83 | 717 | 0.8 | 1 | 66.3 | 13.27 | 0.482 |
| 718 | 0.8 | 100 | 67.7 | 11.96 | 0.778 | 718 | 0.8 | 1 | 66.4 | 12.63 | 0.009 |
| 719 | 0.8 | 100 | 67.5 | 14.605 | 0.775 | 719 | 0.8 | 5 | 67.5 | 12.35 | 0.093 |
| 720 | 0.8 | 100 | 68.9 | 11.035 | 0.812 | 720 | 0.8 | 5 | 67.9 | 11.71 | 0.678 |
| 721 | 0.8 | 100 | 72.7 | 12.52 | 0.802 | 721 | 0.8 | 1 | 72.1 | 12.15 | 0.062 |
| 722 | 0.2 | 100 | 64.2 | 12.175 | 0.843 | 722 | 0.2 | 55 | 63.8 | 11.84 | 0.79 |
| 723 | 0.2 | 100 | 66.9 | 12.22 | 0.821 | 723 | 0.2 | 40 | 66.3 | 12.44 | 0.785 |
| 724 | 0.2 | 100 | 69.7 | 10.355 | 0.85 | 724 | 0.2 | 65 | 69.3 | 10.27 | 0.806 |
| 725 | 0.2 | 100 | 61.1 | 12.97 | 0.834 | 725 | 0.2 | 45 | 60.8 | 11.99 | 0.514 |
| 726 | 0.2 | 100 | 56.3 | 9.89 | 0.824 | 726 | 0.2 | 35 | 56.4 | 9.77 | 0.813 |
| 727 | 0.2 | 100 | 57.3 | 9.895 | 0.825 | 727 | 0.2 | 45 | 57.4 | 10.05 | 0.737 |
| 728 | 0.2 | 100 | 72 | 9.35 | 0.8 | 728 | 0.2 | 40 | 70.9 | 9.91 | 0.789 |
| 729 | 1 | 100 | 62.6 | 9.825 | 0.817 | 729 | 1 | 1 | 62.1 | 10.61 | 0.705 |
| 730 | 1 | 100 | 57.3 | 11.135 | 0.802 | 730 | 1 | 1 | 57.4 | 11.46 | 0.061 |
| 731 | 1 | 100 | 52.8 | 9.985 | 0.798 | 731 | 1 | 0 | 51.4 | 9.51 | 0.304 |
| 732 | 1 | 100 | 68.4 | 11.405 | 0.807 | 732 | 1 | 0 | 68.2 | 10.31 | 0.101 |
| 733 | 1 | 100 | 63.3 | 13.41 | 0.783 | 733 | 1 | 0 | 62.3 | 12.43 | 0.149 |
| 734 | 1 | 100 | 64.5 | 12.57 | 0.744 | 734 | 1 | 0 | 64.6 | 11.91 | 0.471 |
| 735 | 1 | 100 | 68.7 | 11.445 | 0.829 | 735 | 1 | 1 | 68.5 | 12.6 | 0.072 |
| 736 | 0 | 100 | 64.8 | 11.595 | 0.811 | 736 | 0 | 100 | 63.9 | 10.43 | 0.796 |
| 737 | 0 | 100 | 69 | 11.71 | 0.817 | 737 | 0 | 100 | 68.4 | 11.89 | 0.777 |
| 738 | 0 | 100 | 72.5 | 10.93 | 0.835 | 738 | 0 | 100 | 71.3 | 12.17 | 0.822 |
| 739 | 0 | 100 | 74.9 | 11.615 | 0.805 | 739 | 0 | 100 | 74.5 | 12.07 | 0.784 |
| 740 | 0 | 100 | 70.6 | 12.93 | 0.792 | 740 | 0 | 100 | 69.4 | 13.87 | 0.838 |
| 741 | 0 | 100 | 72.1 | 10.79 | 0.818 | 741 | 0 | 100 | 71.6 | 11.79 | 0.829 |
| 742 | 0 | 100 | 69.4 | 10.975 | 0.827 | 742 | 0 | 100 | 69.3 | 11.75 | 0.795 |

**Table S7. 2-week and 3-week post-fire morphology and physiology measurements of *Picea engelmannii* saplings under differing levels of fire intensity.** ID refers to sapling tag number, FRE denotes the fire radiative energy dose (MJ m^-2^), height (cm), PCG denotes the percentage canopy that is green (%), DRC denotes diameter at root collar (mm), and Fv/Fm the chlorophyll fluorescence.

| 2-week post-fire data | | | | | | 3-week post-fire data | | | | | |
| --- | --- | --- | --- | --- | --- | --- | --- | --- | --- | --- | --- |
| ID | FRE | PCG | Height | DRC | Fv/FM | ID | FRE | PCG | Height | DRC | Fv/FM |
| 701 | 0.4 | 20 | 57.8 | 12.37 | 0.634 | 701 | 0.4 | 25 | 58.8 | 10.52 | 0.775 |
| 702 | 0.4 | 25 | 51 | 11.35 | 0.69 | 702 | 0.4 | 15 | 51.1 | 11.1 | 0.72 |
| 703 | 0.4 | 25 | 46.4 | 10.97 | 0.084 | 703 | 0.4 | 15 | 47.9 | 10.21 | 0.755 |
| 704 | 0.4 | 20 | 53.7 | 11.7 | 0.375 | 704 | 0.4 | 25 | 54.4 | 10.02 | 0.775 |
| 705 | 0.4 | 30 | 53.9 | 10.59 | 0.808 | 705 | 0.4 | 30 | 54.8 | 9.19 | 0.818 |
| 706 | 0.4 | 30 | 60.1 | 11.06 | 0.22 | 706 | 0.4 | 25 | 59.8 | 10.89 | 0.819 |
| 707 | 0.4 | 35 | 56 | 13.62 | 0.799 | 707 | 0.4 | 40 | 55.4 | 12.6 | 0.846 |
| 708 | 0.6 | 10 | 51.1 | 9.94 | 0.626 | 708 | 0.6 | 5 | 50.8 | 9.73 | 0.749 |
| 709 | 0.6 | 10 | 54.8 | 10.17 | 0.402 | 709 | 0.6 | 1 | 54.6 | 10.08 | 0.704 |
| 710 | 0.6 | 10 | 50.9 | 14.87 | 0.789 | 710 | 0.6 | 5 | 51.1 | 13.93 | 0.753 |
| 711 | 0.6 | 15 | 57.9 | 14.44 | 0.672 | 711 | 0.6 | 10 | 57.3 | 13.04 | 0.707 |
| 712 | 0.6 | 15 | 56.5 | 11.16 | 0.572 | 712 | 0.6 | 5 | 56.4 | 10.37 | 0.784 |
| 713 | 0.6 | 5 | 64 | 11.25 | 0.63 | 713 | 0.6 | 2 | 65.7 | 10.67 | 0.762 |
| 714 | 0.6 | 0 | 62.4 | 11.33 | 0.06 | 714 | 0.6 | 0 | 62.1 | 10.62 | 0.246 |
| 715 | 0.8 | 5 | 60.3 | 12.36 | 0.473 | 715 | 0.8 | 1 | 59.8 | 12.74 | 0.687 |
| 716 | 0.8 | 10 | 63.1 | 13.41 | 0.453 | 716 | 0.8 | 5 | 65.4 | 12.57 | 0.711 |
| 717 | 0.8 | 0 | 65.4 | 13.31 | 0.387 | 717 | 0.8 | 1 | 66.2 | 13.09 | 0.718 |
| 718 | 0.8 | 1 | 67 | 12.35 | 0.532 | 718 | 0.8 | 1 | 67.3 | 12.92 | 0.781 |
| 719 | 0.8 | 10 | 67.3 | 12.13 | 0.497 | 719 | 0.8 | 5 | 67.4 | 12.75 | 0.729 |
| 720 | 0.8 | 10 | 69.6 | 11.74 | 0.709 | 720 | 0.8 | 5 | 69.1 | 12.11 | 0.572 |
| 721 | 0.8 | 5 | 71.3 | 11.93 | 0.591 | 721 | 0.8 | 0 | 72.5 | 12.3 | 0.732 |
| 722 | 0.2 | 40 | 63.8 | 12.44 | 0.812 | 722 | 0.2 | 45 | 64.4 | 12.16 | 0.792 |
| 723 | 0.2 | 35 | 67.3 | 12.28 | 0.813 | 723 | 0.2 | 30 | 67.1 | 12.22 | 0.828 |
| 724 | 0.2 | 50 | 69.3 | 11 | 0.804 | 724 | 0.2 | 50 | 70.2 | 10.27 | 0.782 |
| 725 | 0.2 | 35 | 59.1 | 12.13 | 0.766 | 725 | 0.2 | 20 | 61.2 | 11.91 | 0.679 |
| 726 | 0.2 | 25 | 56.9 | 9.89 | 0.772 | 726 | 0.2 | 15 | 56.1 | 10.25 | 0.8 |
| 727 | 0.2 | 30 | 56.4 | 10.63 | 0.803 | 727 | 0.2 | 30 | 57 | 9.7 | 0.811 |
| 728 | 0.2 | 45 | 72 | 10.18 | 0.805 | 728 | 0.2 | 25 | 70.9 | 10.1 | 0.793 |
| 729 | 1 | 1 | 61 | 10.47 | 0.066 | 729 | 1 | 1 | 62.1 | 10.09 | 0.718 |
| 730 | 1 | 2 | 55.3 | 11.5 | 0.371 | 730 | 1 | 1 | 57.4 | 11.42 | 0.01 |
| 731 | 1 | 0 | 52.5 | 9.71 | 0.008 | 731 | 1 | 0 | 52.1 | 10.03 | 0.016 |
| 732 | 1 | 0 | 68 | 10.46 | 0.093 | 732 | 1 | 0 | 68.9 | 10.14 | 0.04 |
| 733 | 1 | 0 | 62 | 12.75 | 0.061 | 733 | 1 | 0 | 61.9 | 12.58 | 0 |
| 734 | 1 | 0 | 64.3 | 11.7 | 0 | 734 | 1 | 0 | 64.9 | 11.82 | 0 |
| 735 | 1 | 1 | 68.8 | 12.46 | 0.053 | 735 | 1 | 2 | 68.3 | 12.33 | 0.281 |
| 736 | 0 | 100 | 64 | 10.8 | 0.736 | 736 | 0 | 100 | 64.6 | 10.41 | 0.797 |
| 737 | 0 | 100 | 68.3 | 12 | 0.79 | 737 | 0 | 100 | 68.5 | 11.67 | 0.804 |
| 738 | 0 | 100 | 72 | 13.01 | 0.824 | 738 | 0 | 100 | 72.4 | 11.51 | 0.815 |
| 739 | 0 | 100 | 73.8 | 12.64 | 0.786 | 739 | 0 | 100 | 74.4 | 11.95 | 0.781 |
| 740 | 0 | 100 | 70.4 | 13.66 | 0.823 | 740 | 0 | 100 | 69.8 | 12.89 | 0.827 |
| 741 | 0 | 100 | 70.2 | 12.04 | 0.837 | 741 | 0 | 100 | 71.2 | 11.59 | 0.825 |
| 742 | 0 | 100 | 70.4 | 13.05 | 0.765 | 742 | 0 | 100 | 70.6 | 11.04 | 0.803 |

**Table S8. 4-week and 8-week post-fire morphology and physiology measurements of *Picea engelmannii* saplings under differing levels of fire intensity.** ID refers to sapling tag number, FRE denotes the fire radiative energy dose (MJ m^-2^), height (cm), PCG denotes the percentage canopy that is green (%), DRC denotes diameter at root collar (mm), and Fv/Fm the chlorophyll fluorescence.

| 4-week post-fire data | | | | | | 8-week post-fire data | | | | | |
| --- | --- | --- | --- | --- | --- | --- | --- | --- | --- | --- | --- |
| ID | FRE | PCG | Height | DRC | Fv/FM | ID | FRE | PCG | Height | DRC | Fv/FM |
| 701 | 0.4 | 30 | 59.2 | 10.08 | 0.798 | 701 | 0.4 | 20 | 59.4 | 11.24 | 0.691 |
| 702 | 0.4 | 25 | 51.3 | 11.42 | 0.751 | 702 | 0.4 | 20 | 50.4 | 11.2 | 0.692 |
| 703 | 0.4 | 20 | 47.9 | 10.7 | 0.805 | 703 | 0.4 | 15 | 48.7 | 9.89 | 0.746 |
| 704 | 0.4 | 20 | 53.9 | 10.18 | 0.735 | 704 | 0.4 | 15 | 54.8 | 10.33 | 0.623 |
| 705 | 0.4 | 30 | 55.6 | 9.06 | 0.796 | 705 | 0.4 | 25 | 54.7 | 8.88 | 0.801 |
| 706 | 0.4 | 20 | 59.3 | 10.47 | 0.805 | 706 | 0.4 | 20 | 60.1 | 10.29 | 0.758 |
| 707 | 0.4 | 30 | 55.6 | 11.97 | 0.838 | 707 | 0.4 | 25 | 54.7 | 12.6 | 0.826 |
| 708 | 0.6 | 10 | 51.8 | 9.35 | 0.764 | 708 | 0.6 | 5 | 51.2 | 9.31 | 0.756 |
| 709 | 0.6 | 10 | 55.4 | 9.94 | 0.571 | 709 | 0.6 | 5 | 55.9 | 9.96 | 0.598 |
| 710 | 0.6 | 10 | 50.2 | 12.44 | 0.598 | 710 | 0.6 | 5 | 51.2 | 12.82 | 0.693 |
| 711 | 0.6 | 10 | 57.3 | 13.37 | 0.74 | 711 | 0.6 | 5 | 57.9 | 12.69 | 0.244 |
| 712 | 0.6 | 15 | 56.4 | 11.27 | 0.705 | 712 | 0.6 | 5 | 56.7 | 11.81 | 0.462 |
| 713 | 0.6 | 10 | 65.6 | 11.07 | 0.751 | 713 | 0.6 | 2 | 65.8 | 10.54 | 0.763 |
| 714 | 0.6 | 0 | 63.1 | 10.55 | 0.756 | 714 | 0.6 | 0 | 63.5 | 10.52 | 0.797 |
| 715 | 0.8 | 0 | 60.8 | 12.86 | 0.04 | 715 | 0.8 | 0 | 61.4 | 12.08 | 0.77 |
| 716 | 0.8 | 10 | 65.2 | 12.23 | 0.677 | 716 | 0.8 | 5 | 65.3 | 11.68 | 0.722 |
| 717 | 0.8 | 0 | 65.3 | 12.93 | 0.731 | 717 | 0.8 | 0 | 65.5 | 12.22 | 0.775 |
| 718 | 0.8 | 0 | 67.2 | 12.34 | 0.592 | 718 | 0.8 | 0 | 67.6 | 11.48 | 0.722 |
| 719 | 0.8 | 5 | 67.8 | 12.77 | 0.7 | 719 | 0.8 | 0 | 66.9 | 11.74 | 0.722 |
| 720 | 0.8 | 10 | 69.5 | 11.22 | 0.49 | 720 | 0.8 | 5 | 69.2 | 12.81 | 0.615 |
| 721 | 0.8 | 0 | 72.4 | 11.89 | 0.317 | 721 | 0.8 | 0 | 72.8 | 12.07 | 0.756 |
| 722 | 0.2 | 65 | 63.8 | 11.91 | 0.789 | 722 | 0.2 | 50 | 64.4 | 13.08 | 0.787 |
| 723 | 0.2 | 35 | 66.3 | 14.3 | 0.819 | 723 | 0.2 | 30 | 66.1 | 12.12 | 0.806 |
| 724 | 0.2 | 65 | 69.2 | 10.37 | 0.826 | 724 | 0.2 | 65 | 70.4 | 10.22 | 0.789 |
| 725 | 0.2 | 40 | 60.4 | 14.77 | 0.695 | 725 | 0.2 | 30 | 60.5 | 13.19 | 0.784 |
| 726 | 0.2 | 25 | 56.6 | 10.45 | 0.808 | 726 | 0.2 | 20 | 55.7 | 9.45 | 0.704 |
| 727 | 0.2 | 35 | 56.8 | 10.29 | 0.781 | 727 | 0.2 | 30 | 57.2 | 10.35 | 0.767 |
| 728 | 0.2 | 40 | 71.1 | 9.76 | 0.826 | 728 | 0.2 | 40 | 71.9 | 10.39 | 0.806 |
| 729 | 1 | 1 | 62.2 | 9.95 | 0.568 | 729 | 1 | 0 | 62.3 | 10.28 | 0.336 |
| 730 | 1 | 1 | 56.4 | 11.04 | 0.324 | 730 | 1 | 0 | 58.1 | 11.14 | 0.706 |
| 731 | 1 | 0 | 51.8 | 9.03 | 0.418 | 731 | 1 | 0 | 52.6 | 9.27 | 0.019 |
| 732 | 1 | 0 | 68.4 | 10.97 | 0.03 | 732 | 1 | 0 | 68.1 | 11.39 | 0.507 |
| 733 | 1 | 0 | 63.1 | 12.71 | 0.014 | 733 | 1 | 0 | 62.5 | 13.01 | 0.689 |
| 734 | 1 | 0 | 64.6 | 11.51 | 0 | 734 | 1 | 0 | 63.9 | 11.94 | 0.265 |
| 735 | 1 | 1 | 68.2 | 12.69 | 0 | 735 | 1 | 0 | 68.1 | 12.32 | 0.723 |
| 736 | 0 | 100 | 64.9 | 10.46 | 0.704 | 736 | 0 | 100 | 63.8 | 11.88 | 0.711 |
| 737 | 0 | 100 | 68.4 | 11.58 | 0.781 | 737 | 0 | 100 | 69.4 | 12.25 | 0.763 |
| 738 | 0 | 100 | 71.3 | 11.66 | 0.815 | 738 | 0 | 100 | 72.3 | 12.66 | 0.806 |
| 739 | 0 | 100 | 73.9 | 11.73 | 0.762 | 739 | 0 | 100 | 75.3 | 12.13 | 0.768 |
| 740 | 0 | 100 | 69.9 | 13.06 | 0.78 | 740 | 0 | 100 | 71.3 | 13.28 | 0.742 |
| 741 | 0 | 100 | 72.2 | 11.66 | 0.813 | 741 | 0 | 100 | 71.7 | 11.31 | 0.79 |
| 742 | 0 | 100 | 70.1 | 11.14 | 0.824 | 742 | 0 | 100 | 71.2 | 10.85 | 0.833 |

**Table S9. 16-week and 20-week post-fire morphology and physiology measurements of *Picea engelmannii* saplings under differing levels of fire intensity.** ID refers to sapling tag number, FRE denotes the fire radiative energy dose (MJ m^-2^), height (cm), PCG denotes the percentage canopy that is green (%), DRC denotes diameter at root collar (mm), and Fv/Fm the chlorophyll fluorescence.

| 16-week post-fire data | | | | | | 20-week post-fire data | | | | | |
| --- | --- | --- | --- | --- | --- | --- | --- | --- | --- | --- | --- |
| ID | FRE | PCG | Height | DRC | Fv/FM | ID | FRE | PCG | Height | DRC | Fv/FM |
| 701 | 0.4 | 30 | 58.9 | 9.74 | 0.73 | 701 | 0.4 | 30 | 58.8 | 11.09 | 0.654 |
| 702 | 0.4 | 20 | 51.4 | 11.11 | 0.704 | 702 | 0.4 | 5 | 51.2 | 11.16 | 0.768 |
| 703 | 0.4 | 30 | 49.5 | 10.08 | 0.635 | 703 | 0.4 | 20 | 47.6 | 10.3 | 0.632 |
| 704 | 0.4 | 30 | 53.9 | 9.68 | 0.753 | 704 | 0.4 | 30 | 55.6 | 11 | 0.815 |
| 705 | 0.4 | 40 | 55.6 | 9.14 | 0.775 | 705 | 0.4 | 35 | 55.3 | 9.1 | 0.818 |
| 706 | 0.4 | 30 | 59.4 | 10.6 | 0.781 | 706 | 0.4 | 35 | 61.1 | 10.87 | 0.813 |
| 707 | 0.4 | 45 | 56.7 | 11.59 | 0.81 | 707 | 0.4 | 50 | 55.2 | 12.27 | 0.843 |
| 708 | 0.6 | 5 | 54.1 | 9.25 | 0.2 | 708 | 0.6 | 1 | 51.4 | 9.24 | 0.265 |
| 709 | 0.6 | 1 | 55.7 | 10.24 | 0.408 | 709 | 0.6 | 1 | 55.6 | 9.83 | 0.224 |
| 710 | 0.6 | 1 | 50.8 | 12.3 | 0.16 | 710 | 0.6 | 1 | 50.7 | 12.85 | 0.653 |
| 711 | 0.6 | 1 | 57.9 | 12.59 | 0.435 | 711 | 0.6 | 1 | 57.5 | 13.38 | 0.716 |
| 712 | 0.6 | 1 | 56.6 | 10.73 | 0.581 | 712 | 0.6 | 0 | 56.7 | 10.25 | 0.04 |
| 713 | 0.6 | 0 | 66.7 | 10.26 | 0 | 713 | 0.6 | 0 | 64.8 | 10.77 | 0.433 |
| 714 | 0.6 | 0 | 63.4 | 10.5 | 0 | 714 | 0.6 | 0 | 62.4 | 10.63 | 0.351 |
| 715 | 0.8 | 0 | 61.1 | 12.19 | 0 | 715 | 0.8 | 0 | 60.3 | 12.11 | 0.769 |
| 716 | 0.8 | 0 | 65.1 | 12.36 | 0.094 | 716 | 0.8 | 1 | 64.5 | 11.87 | 0.687 |
| 717 | 0.8 | 0 | 65.4 | 13.14 | 0.087 | 717 | 0.8 | 0 | 65.7 | 14.08 | 0 |
| 718 | 0.8 | 0 | 67.1 | 12.45 | 0 | 718 | 0.8 | 0 | 67.3 | 12.41 | 0.602 |
| 719 | 0.8 | 0 | 66.7 | 12.64 | 0 | 719 | 0.8 | 0 | 67.4 | 13.02 | 0.785 |
| 720 | 0.8 | 5 | 68.4 | 11.93 | 0.397 | 720 | 0.8 | 1 | 68.4 | 12.55 | 0.034 |
| 721 | 0.8 | 1 | 73.1 | 12.16 | 0 | 721 | 0.8 | 0 | 71.8 | 12.07 | 0.662 |
| 722 | 0.2 | 65 | 63.9 | 12.11 | 0.767 | 722 | 0.2 | 45 | 64.2 | 12.78 | 0.775 |
| 723 | 0.2 | 45 | 66.4 | 12.89 | 0.805 | 723 | 0.2 | 40 | 65.5 | 12.73 | 0.843 |
| 724 | 0.2 | 75 | 69.9 | 10.61 | 0.804 | 724 | 0.2 | 65 | 70.2 | 11.51 | 0.822 |
| 725 | 0.2 | 35 | 61.1 | 13.4 | 0.817 | 725 | 0.2 | 40 | 60.9 | 14.51 | 0.826 |
| 726 | 0.2 | 30 | 56.5 | 9.67 | 0.75 | 726 | 0.2 | 10 | 56.4 | 10.42 | 0.777 |
| 727 | 0.2 | 35 | 57.5 | 10.18 | 0.8 | 727 | 0.2 | 35 | 57.9 | 10.99 | 0.732 |
| 728 | 0.2 | 30 | 71.9 | 10.18 | 0.789 | 728 | 0.2 | 30 | 72.1 | 10.5 | 0.831 |
| 729 | 1 | 0 | 62.9 | 10.58 | 0 | 729 | 1 | 0 | 62.4 | 9.86 | 0.057 |
| 730 | 1 | 0 | 57.6 | 10.59 | 0 | 730 | 1 | 0 | 57.8 | 10.81 | 0.039 |
| 731 | 1 | 0 | 52.2 | 8.36 | 0 | 731 | 1 | 0 | 53.2 | 9.48 | 0 |
| 732 | 1 | 0 | 68.2 | 9.94 | 0 | 732 | 1 | 0 | 67.9 | 10.89 | 0 |
| 733 | 1 | 0 | 62.5 | 12.67 | 0 | 733 | 1 | 0 | 62.8 | 13.23 | 0 |
| 734 | 1 | 0 | 64.3 | 11.1 | 0 | 734 | 1 | 0 | 63.6 | 11.06 | 0 |
| 735 | 1 | 0 | 68.1 | 10.87 | 0 | 735 | 1 | 0 | 68.6 | 12.63 | 0 |
| 736 | 0 | 100 | 64.2 | 10.34 | 0.769 | 736 | 0 | 99 | 64.4 | 11.31 | 0.709 |
| 737 | 0 | 100 | 68.6 | 11.77 | 0.677 | 737 | 0 | 100 | 69.2 | 12.72 | 0.737 |
| 738 | 0 | 100 | 70.9 | 11.33 | 0.79 | 738 | 0 | 100 | 72.2 | 13.86 | 0.827 |
| 739 | 0 | 100 | 74.4 | 12.1 | 0.633 | 739 | 0 | 100 | 74.8 | 12.37 | 0.717 |
| 740 | 0 | 99 | 69.2 | 13.3 | 0.793 | 740 | 0 | 99 | 69.1 | 13.47 | 0.703 |
| 741 | 0 | 100 | 70.8 | 11.19 | 0.781 | 741 | 0 | 100 | 70.9 | 11.7 | 0.802 |
| 742 | 0 | 100 | 69.9 | 11.01 | 0.749 | 742 | 0 | 100 | 70.4 | 12.19 | 0.728 |

**Table S10. 24-week and 28-week post-fire morphology and physiology measurements of *Picea engelmannii* saplings under differing levels of fire intensity.** ID refers to sapling tag number, FRE denotes the fire radiative energy dose (MJ m^-2^), height (cm), PCG denotes the percentage canopy that is green (%), DRC denotes diameter at root collar (mm), and Fv/Fm the chlorophyll fluorescence.

| 24-week post-fire data | | | | | | 28-week post-fire data | | | |
| --- | --- | --- | --- | --- | --- | --- | --- | --- | --- |
| ID | FRE | PCG | Height | DRC | Fv/FM | ID | FRE | PCG | Height |
| 701 | 0.4 | 30 | 58.4 | 9.41 | 0.133 | 701 | 0.4 | 0 | Dead |
| 702 | 0.4 | 5 | 50.7 | 11.57 | 0.674 | 702 | 0.4 | 0.263 | Alive |
| 703 | 0.4 | 15 | 46.6 | 10.15 | 0.649 | 703 | 0.4 | 0.687 | Alive |
| 704 | 0.4 | 30 | 56.6 | 9.69 | 0.724 | 704 | 0.4 | 0.812 | Alive |
| 705 | 0.4 | 45 | 59.1 | 9.19 | 0.786 | 705 | 0.4 | 0.807 | Alive |
| 706 | 0.4 | 35 | 59.4 | 10.41 | 0.76 | 706 | 0.4 | 0.786 | Alive |
| 707 | 0.4 | 50 | 55.4 | 13.2 | 0.792 | 707 | 0.4 | 0.831 | Alive |
| 708 | 0.6 | 0 | 50.4 | 9.41 | 0 | 708 | 0.6 | 0 | Dead |
| 709 | 0.6 | 0 | 54.6 | 9.33 | 0 | 709 | 0.6 | 0 | Dead |
| 710 | 0.6 | 0 | 49.8 | 12.86 | 0 | 710 | 0.6 | 0.445 | Alive |
| 711 | 0.6 | 1 | 56.9 | 12.64 | 0.677 | 711 | 0.6 | 0.459 | Alive |
| 712 | 0.6 | 0 | 56.6 | 11.4 | 0 | 712 | 0.6 | 0 | Dead |
| 713 | 0.6 | 0 | 65.2 | 10.29 | 0 | 713 | 0.6 | 0 | Dead |
| 714 | 0.6 | 0 | 63.9 | 10.38 | 0 | 714 | 0.6 | 0.004 | Dead |
| 715 | 0.8 | 0 | 60.4 | 12.36 | 0 | 715 | 0.8 | 0.015 | Dead |
| 716 | 0.8 | 0 | 64.6 | 11.25 | 0 | 716 | 0.8 | 0 | Dead |
| 717 | 0.8 | 0 | 65.9 | 13.57 | 0 | 717 | 0.8 | 0 | Dead |
| 718 | 0.8 | 0 | 67.4 | 12.08 | 0 | 718 | 0.8 | 0.009 | Dead |
| 719 | 0.8 | 1 | 66.5 | 12.67 | 0 | 719 | 0.8 | 0.744 | Alive |
| 720 | 0.8 | 1 | 69.2 | 12.78 | 0 | 720 | 0.8 | 0 | Dead |
| 721 | 0.8 | 0 | 72.4 | 12.12 | 0 | 721 | 0.8 | 0 | Dead |
| 722 | 0.2 | 85 | 70.9 | 12.34 | 0.745 | 722 | 0.2 | 0.823 | Alive |
| 723 | 0.2 | 45 | 68.6 | 14.16 | 0.761 | 723 | 0.2 | 0.811 | Alive |
| 724 | 0.2 | 65 | 74.4 | 11.79 | 0.782 | 724 | 0.2 | 0.84 | Alive |
| 725 | 0.2 | 40 | 61.8 | 13.88 | 0.749 | 725 | 0.2 | 0.792 | Alive |
| 726 | 0.2 | 15 | 56.6 | 11.2 | 0.718 | 726 | 0.2 | 0.738 | Alive |
| 727 | 0.2 | 40 | 57.5 | 10.5 | 0.788 | 727 | 0.2 | 0.842 | Alive |
| 728 | 0.2 | 30 | 73.4 | 11 | 0.815 | 728 | 0.2 | 0.809 | Alive |
| 729 | 1 | 0 | 62.2 | 10.23 | 0 | 729 | 1 | 0 | Dead |
| 730 | 1 | 0 | 56.9 | 10.58 | 0 | 730 | 1 | 0 | Dead |
| 731 | 1 | 0 | 53.6 | 9.52 | 0 | 731 | 1 | 0 | Dead |
| 732 | 1 | 0 | 68.4 | 10.88 | 0 | 732 | 1 | 0 | Dead |
| 733 | 1 | 0 | 62.6 | 15.82 | 0 | 733 | 1 | 0 | Dead |
| 734 | 1 | 0 | 63.8 | 11.9 | 0 | 734 | 1 | 0 | Dead |
| 735 | 1 | 0 | 68.3 | 13.07 | 0 | 735 | 1 | 0 | Dead |
| 736 | 0 | 100 | 64.4 | 11.16 | 0.767 | 736 | 0 | 0.799 | Alive |
| 737 | 0 | 100 | 72.2 | 12.46 | 0.751 | 737 | 0 | 0.759 | Alive |
| 738 | 0 | 100 | 75.3 | 11.91 | 0.767 | 738 | 0 | 0.808 | Alive |
| 739 | 0 | 100 | 76.6 | 13.22 | 0.78 | 739 | 0 | 0.796 | Alive |
| 740 | 0 | 100 | 70.8 | 14.57 | 0.783 | 740 | 0 | 0.808 | Alive |
| 741 | 0 | 100 | 77.1 | 11.94 | 0.766 | 741 | 0 | 0.813 | Alive |
| 742 | 0 | 100 | 70.2 | 12.14 | 0.78 | 742 | 0 | 0.768 | Alive |
